# RAG2 and XLF/Cernunnos interplay reveals a novel role for the RAG complex in DNA repair

**DOI:** 10.1038/ncomms10529

**Published:** 2016-02-02

**Authors:** Chloé Lescale, Vincent Abramowski, Marie Bedora-Faure, Valentine Murigneux, Gabriella Vera, David B. Roth, Patrick Revy, Jean-Pierre de Villartay, Ludovic Deriano

**Affiliations:** 1 Departments of Immunology and Genomes and Genetics, Institut Pasteur, CNRS-URA 1961, Paris 75015, France; 2 Laboratory of Genome Dynamics in the Immune System, INSERM UMR1163, Université Paris Descartes Sorbonne Paris Cité, Institut Imagine, Paris 75015, France; 3 Department of Pathology and Laboratory Medicine, University of Pennsylvania, Philadelphia, Pennsylvania 19104, USA

## Abstract

XRCC4-like factor (XLF) functions in classical non-homologous end-joining (cNHEJ) but is dispensable for the repair of DNA double-strand breaks (DSBs) generated during V(D)J recombination. A long-standing hypothesis proposes that, in addition to its canonical nuclease activity, the RAG1/2 proteins participate in the DNA repair phase of V(D)J recombination. Here we show that in the context of RAG2 lacking the C-terminus domain (*Rag2*^*c/c*^ mice), XLF deficiency leads to a profound lymphopenia associated with a severe defect in V(D)J recombination and, in the absence of p53, increased genomic instability at V(D)J sites. In addition, *Rag2*^*c/c*^ *XLF*^*−/−*^ *p53*^*−/−*^ mice develop aggressive pro-B cell lymphomas bearing complex chromosomal translocations and gene amplifications involving *Igh* and *c-myc*/*pvt1* loci. Our results reveal an unanticipated functional interplay between the RAG complex and XLF in repairing RAG-induced DSBs and maintaining genome integrity during antigen receptor gene assembly.

## Abstract (web summary)

Antigen receptor diversity relies on careful DNA cleavage and repair. Here the authors identify a functional interplay between RAG2 and XLF during V(D)J recombination, revealing an important role for the RAG complex in repairing induced DNA double-strand breaks and maintaining genome integrity.

## Graphical abstract

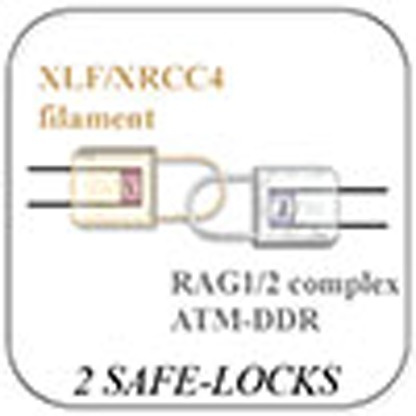

B and T lymphocyte development relies on the assembly of immunoglobulin (Ig) and T-cell receptor (TCR) variable exons from variable (V), diversity (D) and joining (J) gene segments via a cut-and-paste mechanism termed V(D)J recombination^1^. This process occurs in developing lymphocytes during the G1 phase of the cell cycle, and is initiated when the recombination-activating gene products RAG1 and RAG2 (forming the RAG endonuclease) introduce double-strand breaks (DSBs) between V, D or J coding gene segments and flanking recombination signal sequences (RSSs)^2^. RAG-mediated cleavage at a pair of RSSs generates four broken DNA ends: two blunt 5′ phosphorylated signal ends, which terminate in the RSS, and two covalently sealed (hairpin) coding ends. After cleavage, the RAG proteins stay associated with the DNA ends in a so-called post-cleavage complex (PCC)^2^. Subsequently, the classical non-homologous end-joining pathway (cNHEJ) joins these DNA ends in a recombinant configuration, forming a coding joint (CJ) (the rearranged antigen receptor gene) and a reciprocal signal joint^3^,^4^,^5^.

RAG-induced DNA breaks activate the Ataxia telangiectasia mutated (ATM) kinase-dependent DNA damage response (DDR)^6^. ATM-dependent p53 phosphorylation mediates the G1/S checkpoint that arrests or eliminates cells with unrepaired DSBs. ATM also phosphorylates chromatin- and/or DNA-associated proteins, including the histone variant H2AX (forming γH2AX), p53 binding protein 1 (53BP1), mediator of DNA damage checkpoint 1 (MDC1) and factors of the MRE11 complex (MRE11, RAD50 and NBS1) that assemble over large DNA regions of the chromatin on both sides of DNA breaks to form so-called nuclear DNA repair foci. ATM-dependent DDR, beyond activating checkpoints, may contribute to DSB repair through stabilization of DNA ends. Because the stabilization function of ATM depends on its kinase activity, formation of ATM-dependent DNA repair foci has been proposed to stabilize/tether DNA ends for proper joining via cNHEJ. In fact, in ATM-deficient cells undergoing V(D)J recombination, a fraction of coding ends evade from the PCC and are occasionally joined aberrantly forming hybrid joints (HJs) (atypical nonproductive rearrangements formed by the ligation of a signal end to a coding end) or chromosomal deletions, inversions and translocations^6^,^7^. Despite these defects, ATM-deficient cells are still able to perform robust V(D)J recombination and ATM-deficient mice are only moderately immune-deficient. Similarly, deficiency of H2AX or 53BP1 has no demonstrable effect on the repair of RAG-mediated DSBs, indicating that other accessory proteins might compensate impaired ATM-DDR functions during the repair of RAG-DNA breaks^6^,^8^.

During cNHEJ repair^3^, the Ku70/80 heterodimer (Ku) binds DNA ends and recruits the DNA-dependent protein kinase catalytic subunit (DNA-PKcs) to form the DNA-PK holoenzyme. DNA-PK phosphorylates multiple substrates, promoting synapsis of DNA ends and facilitating the recruitment of end processing and ligation enzymes. One such enzyme is the Artemis endonuclease that carries out hairpin opening at coding ends and prepares them for joining. Finally, the XRCC4-like factor (XLF)-Ligase IV complex performs ligation of DNA ends. In mice, deficiency for cNHEJ factors (except XLF, see below) results in severe combined immunodeficiency owing to the inability to complete repair of RAG-DNA breaks. In addition, cNHEJ-deficient mice that are also deficient for p53 characteristically develop pro-B cell lymphomas harbouring RAG-dependent translocations and gene amplification involving the *Igh* and *c-myc* loci (or *n-myc* in the case of Artemis deficiency)^3^,^4^,^5^.

XLF (also called Cernunnos or NHEJ1) was identified through both cDNA complementation of cells derived from an IR-sensitive immunodeficient patient^9^ and through a yeast two-hybrid screen for XRCC4-interacting partners^10^. XLF and XRCC4 are two distantly related members of the same protein family and share structural similarity^11^,^12^,^13^. Together, they form long filaments, thought to help DNA end tethering during repair^14^,^15^,^16^,^17^,^18^. In contrast to other cNHEJ-deficient mice, XLF-deficient mice are not markedly immune-deficient and pro-B cell lines derived from these animals perform nearly normal V(D)J recombination^19^,^20^. In addition, XLF/p53-deficient animals, unlike other cNHEJ/p53 double-deficient mice, rarely die of pro-B cell lymphomas but instead develop T-cell lymphomas characteristic of p53 deficiency^20^. These results are consistent with normal overall V(D)J recombination in developing XLF-deficient lymphocytes and led to the speculation that lymphocyte-specific factors/pathways compensate for XLF function during V(D)J recombination^3^,^19^,^20^. This is supported by analysis of V(D)J recombination in cells deficient for both ATM-dependent DDR and XLF^8^. A combined deficiency of ATM, 53BP1 or H2AX with XLF deficiency results in a severe block in lymphocyte development with a significant defect in the repair of RAG-mediated DSBs, indicating functional redundancy between XLF and ATM-DDR factors during V(D)J recombination^8^,^21^. Importantly, although single deficiencies for XLF or individual DDR factors generally have little effect on V(D)J recombination, these deficiencies have been demonstrated to have a profound effect on non-V(D)J cNHEJ-mediated DSB repair (for example, irradiation or activation-induced cytidine deaminase (AID)-induced DSBs during the process of class switch recombination)^8^,^22^, indicating that such compensatory functions are specific to the repair of RAG-mediated DNA breaks. The nature of the mechanism(s) underlying these discrepancies is unknown.

One major difference between genotoxic- or AID-induced DSBs and those occurring during V(D)J recombination is the participation of the RAG complex in the latter^3^,^23^. After cleavage *in vitro*, the RAG proteins remain associated with the DNA ends in a PCC, providing evidence for a potential layer of regulation at the joining step^2^. Separation of function mutants of RAG1 and RAG2 that are capable of cleavage *in vitro* but exhibit defects in performing V(D)J recombination of episomal substrates *in vivo* provided further evidence that the RAG PCC might serve a crucial function in joining coding and signal ends^23^,^24^,^25^,^26^,^27^. In addition, studies showed that certain RAG mutations that cause destabilization of the RAG PCC *in vitro* correlates with abnormal repair by homologous recombination and alternative NHEJ *in vivo*^3^,^28^,^29^,^30^,^31^,^32^. These results led to a model in which the quite stable RAG PCC, through an unknown mechanism, shepherds DNA ends to the cNHEJ machinery for repair, thus protecting them from error-prone end-joining pathways and aberrant recombination events. In that regard, a truncated RAG2 lacking the C-terminus (core RAG2: amino acids 1-352 (refs 33, 34)) is of particular interest. The RAG2 C-terminus contains a flexible acidic hinge, a plant homeodomain capable of recognizing histone H3K4 trimethylation and a cell cycle-regulated protein degradation signal^35^. While dispensable for overall V(D)J recombination, this region is important for V(D)J recombination efficiency and fidelity^34^,^35^,^36^,^37^,^38^. Core RAG2 destabilizes the RAG-PCC *in vitro* and increases the rate of aberrant recombination product formation *in vivo*^29^,^30^,^31^,^36^,^37^,^39^. Additionally, core RAG2 knockin/p53-deficient mice develop aggressive thymic lymphomas bearing complex translocations involving *Tcr* and *Ig* loci^30^, a phenotype reminiscent of ATM-deficient mice^7^,^40^. These results suggested that comparable RAG-PCC defects might underlie genomic instability and lymphomagenesis in *Rag2*^*c/c*^ *p53*^*−/−*^ and *Atm*^*−/−*^ mice^30^. Altogether, these biochemical, genetic and *in vivo* studies have raised the intriguing possibility that, in addition to their primary role in DNA cleavage at RSSs, the RAG1 and RAG2 proteins might be implicated in the joining step of V(D)J recombination. To explain the paradoxical observation that we and others have made on the normal V(D)J recombination in *XLF*^*−/−*^ mice^19^,^20^, we hypothesized a direct role for the RAG proteins in the repair of chromosomal RAG-DSBs^3^,^19^. In this study, we identify overlapping functions between the RAG complex and XLF in V(D)J recombination, lymphocyte development and maintenance of genome stability.

## Results

### Core RAG2/XLF-deficient mice are lymphopenic

**Figure 1:**
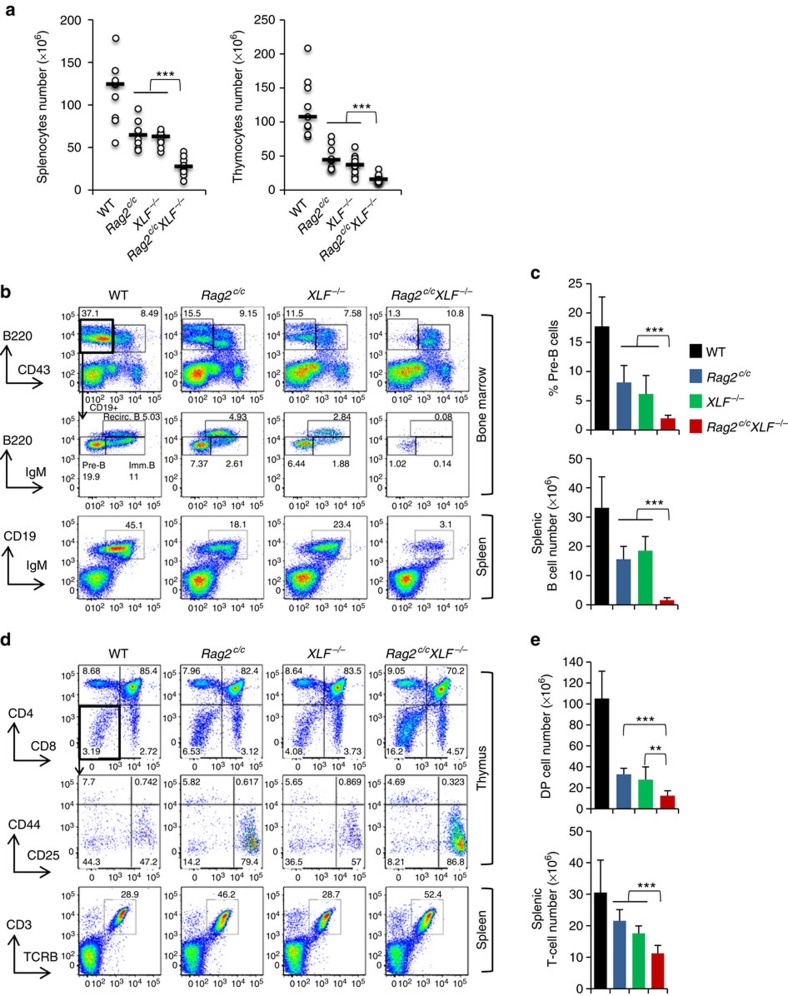
Lymphopenia associated with severe B cell developmental block in *Rag2*^*c/c*^ *XLF*^*−/−*^ mice. Lymphocyte development in WT, *Rag2*^*c/c*^, *XLF*^*−/−*^ and *Rag2*^*c/c*^ *XLF*^*−/−*^ mice. (**a**) Total splenocyte (left) and thymocyte (right) numbers. Each dot represents one mouse and bars indicate the median. (**b**,**c**) Analysis of B cell development. (**b**) Representative FACS analysis of bone marrow and spleen using B cell markers. Numbers on plots represent percentages of total cells. (**c**) Percentage of bone marrow CD43^−^B220^lo^CD19^+^IgM^−^ pre-B cells (upper) and total number of splenic CD19^+^IgM^+^ B cells (bottom). (**d**,**e**) Analysis of T-cell development. (**d**) Representative FACS analysis of thymus and spleen using T-cell markers. (**e**) Total number of thymic DP cells (upper) and splenic CD3^+^TCRβ^+^ T cells (bottom). Histograms represent means±s.d. from at least five mice of each genotype. **0.001≤*P*<0.01, ***P<0.001.

To elucidate whether the RAG complex has overlapping functions with XLF and/or ATM during lymphocyte development, we bred *Rag2*^*c/c*^ mice^30^,^33^,^34^ with *XLF*^*−/−*^ mice^19^ and *Atm*^*−/−*^ mice^30^,^41^ to generate *Rag2*^*c/c*^ *XLF*^*−/−*^ mice and *Rag2*^*c/c*^ *Atm*^*−/−*^ mice. Consistent with previous reports^19^,^20^,^21^,^30^,^34^, *Rag2*^*c/c*^, *XLF*^*−/−*^ and *Atm*^*−/−*^ mice displayed only partial blocks in B and T lymphopoiesis associated with a reduction in splenocyte numbers (Fig. 1 and Supplementary Fig. 1). In sharp contrast, *Rag2*^*c/c*^ *XLF*^*−/−*^ mice had extremely low numbers of splenocytes associated with an almost complete absence of mature CD19^+^IgM^+^ splenic B cells (Fig. 1b,c) and significantly fewer mature CD3^+^TCRβ^+^ splenic T cells (Fig. 1d,e). Analysis of bone marrow B-cell development in *Rag2*^*c/c*^ *XLF*^*−/−*^ mice revealed a severe block at the CD43^+^B220^low^CD19^+^IgM^−^ progenitor (pro-) B-cell stage during which V(D)J recombination is initiated, as shown by the near absence of CD43^−^B220^low^CD19^+^IgM^−^ precursor (pre-) B cells and IgM^+^ B cells (Fig. 1b,c). *Rag2*^*c/c*^ *XLF*^*−/−*^ mice also had very low numbers of total thymocytes (Fig. 1a), associated with a significant increase in the percentage of CD4^−^CD8^−^ double-negative cells that initiate V(D)J recombination (Fig. 1d). In contrast to B cells, T-cell development was not completely abolished in *Rag2*^*c/c*^ *XLF*^*−/−*^ mice (Fig. 1d,e). This “leaky” T-cell phenotype was previously observed in *XLF*^*−/−*^ *Atm*^*−/−*^ mice^21^ and other cNHEJ-deficient mice^42^,^43^. Interestingly, in contrast to *Rag2*^*c/c*^ *XLF*^*−/−*^ mice, *Rag2*^*c/c*^ *Atm*^*−/−*^ mice displayed almost normal B- and T-cell development and lymphoid organ cellularity as compared with single mutant mice (Supplementary Fig. 1). Thus, although the combined deficiency in RAG2 and ATM does not severely affect B- and T-cell differentiation, core RAG2/XLF deficiency severely compromises lymphocyte differentiation at a progenitor cell stage.

### Impaired *Igk* rearrangement in *Rag2* ^*c/c*^ *XLF* ^*−/−*^ pro-B cells

**Figure 2:**
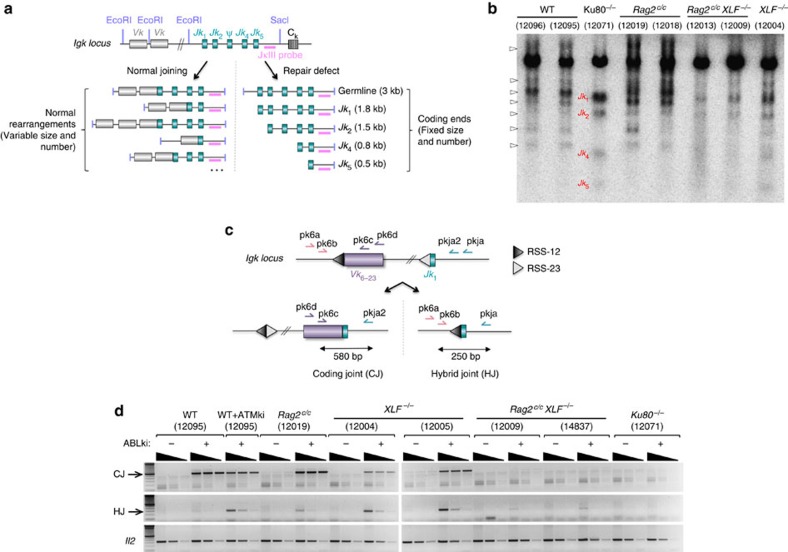
Impaired rearrangement at the endogenous *Igk* locus in *Rag2*^*c/c*^ *XLF*^*−/−*^ B cells. (**a**) Schematic representation of the *Igk* locus. The relative positions of the EcoRI and SacI restriction sites and the JKIII probe are shown. Schematic representations of SacI/EcoRI digest fragments for normal *Vk-Jk* rearrangements (left) and the *Jk1*, *Jk2*, *Jk4*, and *Jk5* coding ends (CE) (right) are represented. SacI/EcoRI digest fragments from *Vk-Jk* rearrangements are variable in size depending on the *Vk* and *Jk* gene segments used and the relative positions of EcoRI/SacI sites, whereas the size of *Jk1*, *2*, *4* and *5* coding ends fragments are 1.8, 1.5, 0.8 and 0.5 kb, respectively. (**b**) Southern blot (using JKIII probe) of EcoRI/SacI digested DNA from *v-abl* pro-B cell lines treated for 72 h with ABLki (see also Supplementary Fig. 2). Arrows indicate normal rearrangements. *Jk*_*1–5*_ CEs are indicated in red. (**c**) Schematic representation of the *Igk* locus with position of primers (arrows) used to assay CJ and HJ formation during inversional *IgkV*_*6–23*_*-J*_*1*_ rearrangement. (**d**) Semi-quantitative nested PCR analysis of *IgkV*_*6–23*_*-J*_*1*_ CJ and HJ from indicated *v-abl* abl pro-B cell lines treated for 72 h with ABLki with or without ATMki. *Il2* gene PCR was used as a loading control. The data presented are representative of at least two experiments using independent cell lines.

To identify the cause of impaired lymphoid development in *Rag2*^*c/c*^ *XLF*^*−/−*^ mice, we analyzed V(D)J recombination in viral-Abelson kinase (*v-abl*) transformed pro-B cell lines generated from wild type (WT), *Rag2*^*c/c*^, *XLF*^*−/−*^, *Rag2*^*c/c*^ *XLF*^*−/−*^ and Ku80-deficient (*Ku80*^*−/−*^) mice. Treatment of *v-abl* transformed pro-B lines with a *v-abl* kinase inhibitor (PD180970 or STI571, hereafter named ABLki) leads to G1 cell cycle arrest, the rapid induction/stabilization of RAG1/2 gene expression and rearrangement of the *Igk* locus or any introduced V(D)J recombination reporter substrate^7^,^44^. Induction of RAG in WT, *Rag2*^*c/c*^, and to a lesser extent *XLF*^*−/−*^ pro-B cells triggered *Vk*-to-*Jk* rearrangement at the endogenous *Igk* locus, as evidence by Southern blotting revealing hybridizing bands of diverse sizes from rearrangements between ∼150 *Vk* gene segments and the four functional *Jk* gene segments from the mouse locus (Fig. 2a,b and Supplementary Fig. 2) and by PCR analysis of inversional *IgkV*_*6–23*_*-J*_*1*_ revealing CJ formation (Fig. 2c,d). Induction of RAG in *Ku80*^*−/−*^ pro-B cells led to the accumulation of unrepaired coding ends at the four *Jk* segments (Fig. 2a,b and Supplementary Fig. 2) and absence of *Vk*_*6–23*_*-Jk*_*1*_ CJs (Fig. 2c,d) as a result of the function of Ku in coding and signal joint formation^45^. Notably, nested PCR amplification of inversional *IgkV*_*6–23*_*-J*_*1*_ rearrangement revealed the formation of HJs in WT cells treated with the ATM kinase-specific inhibitor KU-55933 (ATMki) and in *Rag2*^*c/c*^ cells (Fig. 2c,d), consistent with a role for the RAG complex and ATM in stabilizing cleaved DNA ends^7^,^30^. Strikingly, HJs were also readily detected in *XLF*^*−/−*^ cells (Fig. 2c,d), indicating that although XLF is dispensable for V(D)J recombination, it might participate in the stabilization of RAG-induced DSBs. In *Rag2*^*c/c*^ *XLF*^*−/−*^ pro-B cells, Southern blotting revealed faint hybridizing bands of sizes corresponding to unrepaired coding ends (Fig. 2a,b and Supplementary Fig. 2) and PCR amplification revealed an almost complete lack of CJ and HJ formation (Fig. 2c,d). These results suggest a specific end-joining defect in the absence of functional RAG2 and XLF during V(D)J recombination.

### *Rag2* ^*c/c*^ *XLF* ^*−/−*^ pro-B cells accumulate DNA damage foci

**Figure 3:**
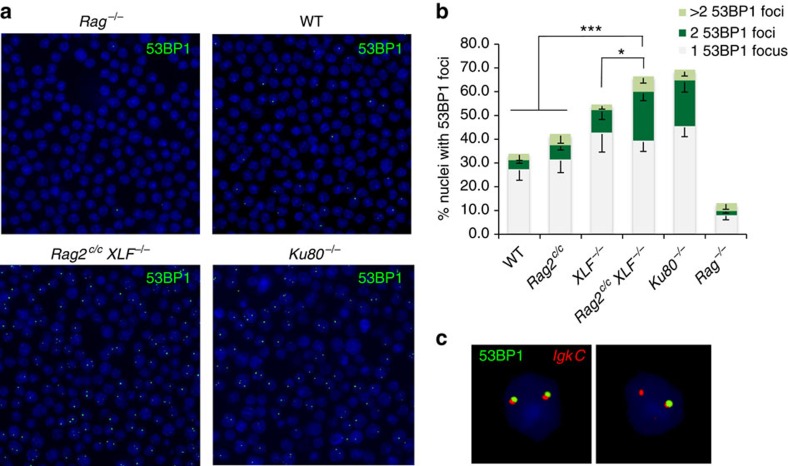
Accumulation of RAG-induced 53BP1 foci in *Rag2*^*c/c*^ *XLF*^*−/−*^ B cells. (**a**) Representative 3D projections of 53BP1 immuno-staining conducted on ABLki-treated *v-abl* pro-B cells. (**b**) Percentage of *v-abl* pro-B cells harbouring 1, 2 or >2 53BP1 foci 65 h post-ABLki treatment. Histograms represent means±s.d. from four independent experiments with two independent cell lines for each genotype (see Supplementary Table 1 for details). *0.01≤*P*<0.05, ***P<0.001. (**c**) Fluorescent light microscopy images of immuno-3D FISH analysis conducted on ABLki-treated *Rag2*^*c/c*^ *XLF*^*−/−*^ *v-abl* pro-B cells. Nuclei were stained with anti-53BP1 antibody (green) and subsequently hybridized with the centromeric *Igk* BAC probe (*Igk C*, red). Images show 2 nuclei with coincident 53BP1/*Igk* signals (see Supplementary Fig. 4 for details).

Although the absence of normal *Vk*-to-*Jk* rearrangement in *Rag2*^*c/c*^ *XLF*^*−/−*^ *v-abl* pro-B cells could be attributed to defects in DSB repair, it could also arise from decreased RAG cleavage^34^. To test for this possibility, we used automated three-dimensional (3D) microscopy to quantify the presence of DNA damage-associated protein (53BP1) foci at the *Igk* locus in G1-arrested pro-B cells^46^,^47^. Upon treatment with ABLki, we found that 33.4% (*n*=36,618 total nuclei analysed) of WT pro-B cells showed intense 53BP1 foci; the majority contained a single distinct spot, although cells were occasionally found to contain two, and less frequently, three or more foci (Fig. 3a,b and Supplementary Table 1). Dual immuno-staining and DNA fluorescent *in situ* hybridization (FISH) revealed that the majority of 53BP1 foci co-localized with phosphorylated H2AX (γH2AX) and with the *Igk* locus, indicating that 53BP1 foci mark the *Igk* locus undergoing RAG-mediated breaks (Fig. 3c and Supplementary Figs 3 and 4). To further assess the specificity of 53BP1 foci in marking RAG-initiated DNA damage, we generated Rag2-deficient (*Rag2*^*−/−*^) pro-B cell lines, that are unable to initiate RAG DNA breaks^48^. In ABLki-treated *Rag2*^*−/−*^ cell lines, 53BP1 foci were detected in only 12.7% (*n*=18,158) of the cells, consistent with the absence of V(D)J rearrangement in these cells^48^, whereas 53BP1 foci were detected in 68% (*n*=14,423) of *Ku80*^*−/−*^ cells (Fig. 3a,b and Supplementary Table 1). Strikingly, we found that, similarly to Ku80 deficiency, 65.9% (*n*=38,875) of *Rag2*^*c/c*^ *XLF*^*−/−*^ pro-B cells harboured 53BP1 foci (Fig. 3a,b and Supplementary Table 1), indicating that DNA breaks are readily formed in core RAG2/XLF-deficient cells. Also, similar to Ku80-deficient pro-B cells, 20.4% of *Rag2*^*c/c*^ *XLF*^*−/−*^ cells contained two 53BP1 foci as compared with 3.6% (*n*=36,618), 6% (*n*=46,264) and 9.4% (*n*=34,184) in WT, *Rag2*^*c/c*^ and *XLF*^*−/−*^ pro-B cells, respectively (Fig. 3a,b and Supplementary Table 1). Overall, these results indicate that the combined RAG2 and XLF deficiency strongly impairs repair of RAG-mediated DNA breaks, similar to what is seen in the absence of general cNHEJ factors.

### End-joining defects in *Rag2* ^*c/c*^ *XLF* ^*−/−*^ cells

**Figure 4:**
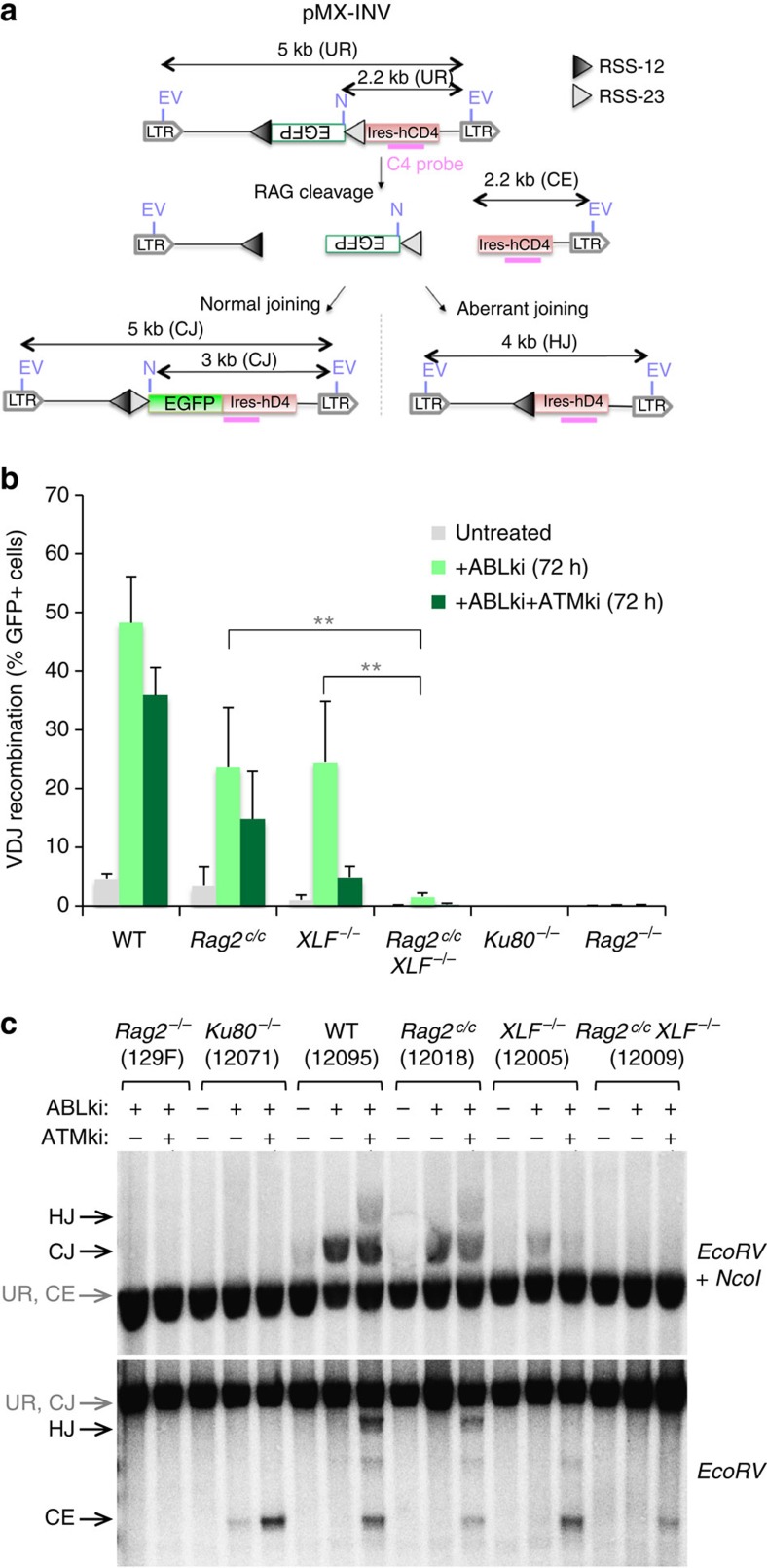
Defective inversional V(D)J recombination in *Rag2*^*c/c*^ *XLF*^*−/−*^ B cells. (**a**) Schematic representation of pMX-INV recombination substrate. The 12-recombination signal sequence (RSS-12; black triangle), GFP cDNA, 23-recombination signal sequence (RSS-23; grey triangle), IRES—human CD4 cDNA (IRES-hCD4), LTRs, EcoRV (EV) sites, NcoI (N) site, C4 probe (pink bar), and the expected sizes for the un-rearranged substrate (UR), coding end intermediates (CE), coding joints (CJ) and hybrid joints (HJ) are indicated. (**b**) *v-abl* pro-B cell lines treated for 72 h with ABLki with or without ATM kinase inhibitor (ATMki) were assayed for pMX-INV rearrangement by flow cytometry, with the percentage of GFP expressing cells indicated. Histograms represent the means±s.d. of 2 WT (12095 and 12096), 2 *Rag2*^*c/c*^ (12018 and 12019), 4 *XLF*^*−/−*^ (12004, 12005, 16218 and 16488), 3 *Rag2*^*c/c*^ *XLF*^*−/*−^ (12009, 12013 and 14837), 1 *Ku80*^*−/−*^ (12071) and 3 *Rag2*^*−/−*^ (129, 16605 and 16610) independent cell lines. Experiments were repeated three times for ABLki-treated cells and performed once for ABLki/ATMki-treated cell lines. **P<0.005. (**c**) The indicated *v-abl* pro-B cell lines containing the pMX-INV substrate were treated for 72 h with ABLki with or without ATMki and assayed by Southern blotting; EcoRV/NcoI digest—C4 probe (top panel) and EcoRV digest—C4 probe (bottom panel). The data presented are representative of at least two experiments using independent cell lines (see also Supplementary Fig. 7).

To test unequivocally for V(D)J recombination defects, we transduced *v-abl* pro-B cell lines from each genotype with the pMX-RSS-GFP/IRES-hCD4 retroviral recombination substrate (pMX-INV) that allows for GFP expression upon successful chromosomal inversional RAG-mediated recombination and for assessment of the rearrangement status and recombination intermediates by Southern blot analysis^7^,^34^ (Fig. 4a). Flow cytometry and CJ product analyses showed robust levels of pMX-INV rearrangement in ABLki-treated WT, *Rag2*^*c/c*^, and *XLF*^*−/−*^ cells (Fig. 4b,c and Supplementary Figs 5 and 6a), as expected^19^,^20^,^34^. Strikingly, pMX-INV rearrangement was severely impaired in *Rag2*^*c/c*^ *XLF*^*−/−*^ cells as compared with WT (30-fold decrease, *P*<0.005), *Rag2*^*c/c*^ (15-fold decrease, *P*<0.005) and *XLF*^*−/−*^ (15-fold decrease, *P*<0.005) cells (Fig. 4b and Supplementary Fig. 5b), leading to the absence of rearrangement products (CJ and HJ) (Fig. 4c and Supplementary Fig. 6). In agreement with *in vivo* results (Supplementary Fig. 1), pMX-INV rearrangement was not dramatically affected in *Rag2*^*c/c*^ pro-B cells treated with the ATMki or in *Rag2*^*c/c*^ *Atm*^*−/−*^ pro-B cells as compared with *Rag2*^*c/c*^ and *Atm*^*−/−*^ cells (Fig. 4 and Supplementary Figs 5b and 7). Treatment of WT and *Rag2*^*c/c*^ pro-B cells with ATMki led to the accumulation of HJs and coding ends (CEs) (Fig. 4c and Supplementary Fig. 6), as expected^7^. In contrast, rearrangement was almost completely abolished in *XLF*^*−/−*^ cells treated with the ATMki, leading to the accumulation of CEs instead of CJ products (Fig. 4c and Supplementary Fig. 6). These results are in accord with the previously described redundancy between XLF and ATM in joining DNA breaks during V(D)J recombination^8^,^21^. Moreover, unlike ATMki-treated *XLF*^*−/−*^ cells and *Ku80*^*−/−*^ cells, very little, if any, CEs accumulated in *Rag2*^*c/c*^ *XLF*^*−/−*^ cells (Fig. 4c and Supplementary Fig. 6). The results are consistent with the weak accumulation of coding ends observed at the *Igk* locus in *Rag2*^*c/c*^ *XLF*^*−/−*^ pro-B cells (Fig. 2 and Supplementary Fig. 2). Lack of CJ in the absence of CE accumulation has been previously reported in *H2AX*^*−/−*^ *XLF*^*−/−*^ and *53BP1*^*−/−*^ *XLF*^*−/−*^ cells because H2AX and 53BP1 are required to protect unrepaired CEs from ATM-mediated degradation^8^,^21^,^49^. To test whether RAG2 has a similar function in preventing CE degradation, we performed pMX-INV recombination assays in *Rag2*^*c/c*^ *XLF*^*−/−*^ cells treated with ATMki. In ABLki-treated *Rag2*^*c/c*^ *XLF*^*−/−*^ cells, ATM kinase inhibition led to the appearance of a visible CE band, indicating that combined RAG2 and XLF deficiency leads to accumulation of unrepaired coding ends that are exposed to ATM-dependent degradation (Fig. 4c and Supplementary Fig. 6). In support to these results, we also readily detected *IgkJ*_*1–4*_ CEs in *Rag2*^*c/c*^ *XLF*^*−/−*^ cells treated with ABLki and ATMki (Supplementary Fig. 2b). Interestingly, we also noticed that ABLki/ATMki treated *Ku80*^*−/−*^ cells showed stronger levels of unrepaired CEs as compared with ABLki-treated *Ku80*^*−/−*^ cells, indicating that a significant fraction of unrepaired CEs are subjected to ATM-dependent end degradation in Ku80-deficient cells (Fig. 4c).

**Figure 5:**
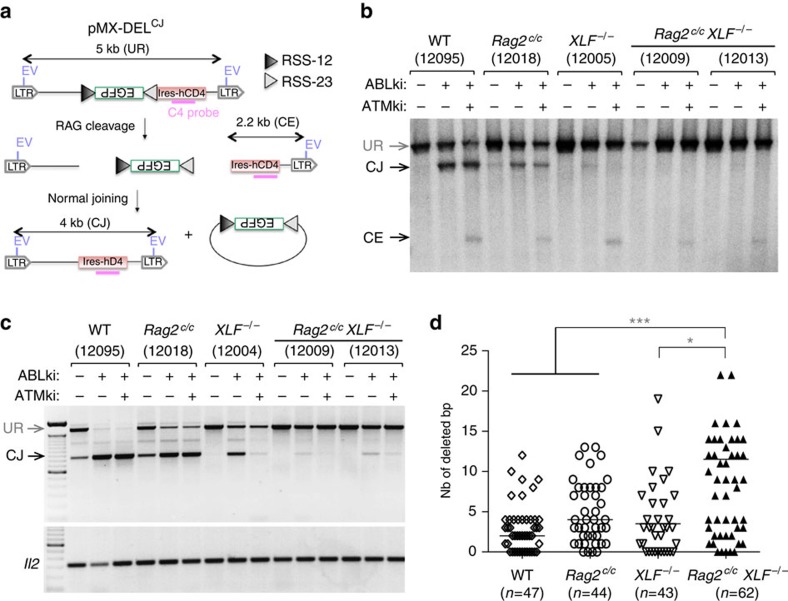
Defective deletional V(D)J recombination and abnormal CJ formation in *Rag2*^*c/c*^ *XLF*^*−/−*^ B cells. (**a**) Schematic representation of pMX-DEL^CJ^ recombination substrate with components, intermediates and products as defined for pMX-INV in Fig. 4a. (**b**) *v-abl* pro-B cell lines containing pMX-DEL^CJ^ were treated for 72 h with ABLki with or without ATMki and assayed by Southern blotting; EcoRV digest—C4 probe. Experiments were repeated at least two times. (**c**) PCR analysis of pMX-DEL^CJ^ CJs from indicated *v-abl* abl pro-B cell lines treated for 72 h with ABLki with or without ATMki. *Il2* gene PCR was used as a loading control. Experiments were repeated at least two times and PCR specificity was confirmed by Southern blotting (Supplementary Fig. 8). (**d**) Sanger sequencing of PCR amplified CJs from *v-abl* pro-B cells (see also Supplementary Table 2). Number of base-pair deletions is indicated on the *y*-axis. The horizontal bar represents the median. The total number of sequenced CJs (*n*) is indicated. Outliers (>25 bp deletion) are not shown but are included in calculation of the median. *0.01≤*P*<0.05, ***P<0.001.

We also tested for V(D)J recombination defects with a substrate that rearranged by deletion (pMX-DEL-CJ), resulting in the formation of a CJ that remained within the chromosomal context (Fig. 5a and Supplementary Fig. 8a)^7^. PCR and Southern blot analysis confirmed severely defective end-joining in ABLki-treated *Rag2*^*c/c*^ *XLF*^*−/−*^ pro-B cell lines and a marked increase in un-joined CEs upon treatment with ATMki (Fig. 5b,c and Supplementary Fig. 8b). Notably, PCR analysis of CJ products revealed that ATM inhibition slightly, but consistently, reduced CJ formation in *Rag2*^*c/c*^ *XLF*^*−/−*^ cells, highlighting a RAG-independent function of ATM in XLF-deficient cells (Fig. 5c and Supplementary Fig. 8b). These results were also supported by the reduced GFP expression in ABLki/ATMki treated pMX-INV *Rag2*^*c/c*^ *XLF*^*−/−*^ cells as compared with ABLki treatment only (Fig. 4b and Supplementary Fig. 5b). Sequences of rare CJs derived from *Rag2*^*c/c*^ *XLF*^*−/−*^ cells harboured increased deletions and microhomology usage as compared with single mutant and WT cells (Fig. 5d, Supplementary Fig. 9 and Supplementary Table 2). These events were reminiscent of rare joints seen in cNHEJ-deficient cells, indicating aberrant alternative NHEJ in core RAG2/XLF-deficient cells. Together, these results indicate that the RAG complex and XLF participate in repairing RAG-mediated DNA breaks and in protecting them from aberrant end-joining and ATM-mediated degradation.

### Genomic instability in *Rag2* ^*c/c*^ *XLF* ^*−/−*^ *p53* ^*−/−*^ B cells

**Figure 6:**
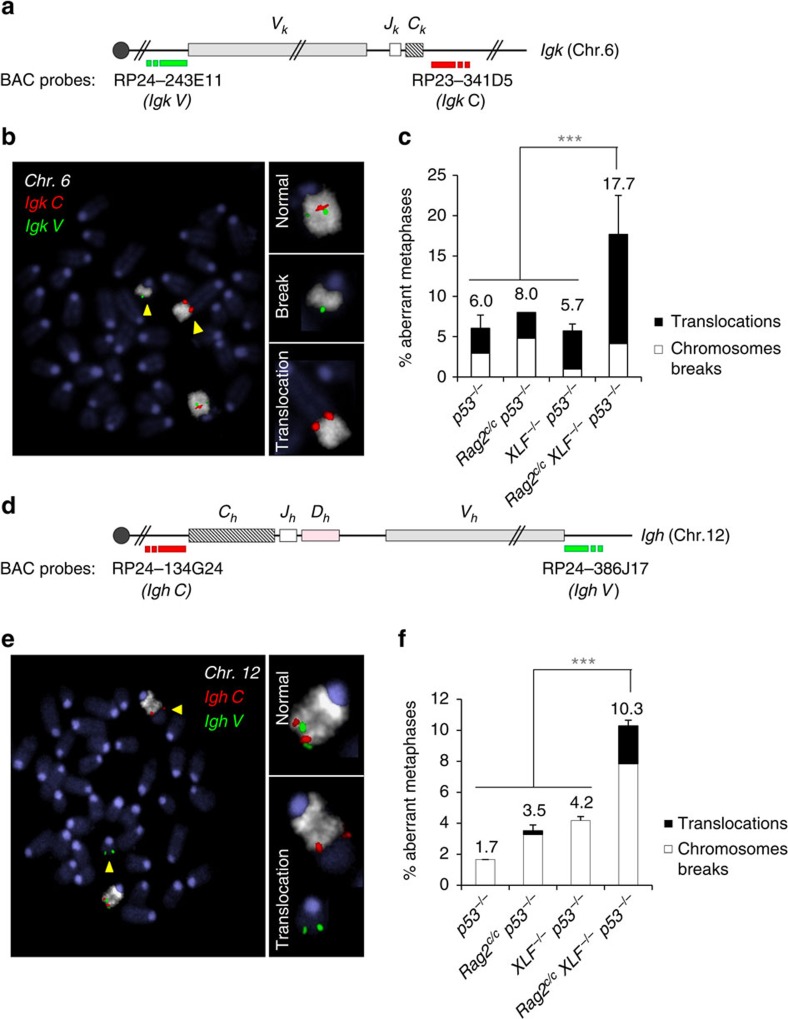
Aberrant V(D)J recombination leads to genomic instability in *Rag2*^*c/c*^ *XLF*^*−/−*^ *p53*^*−/−*^ B cells. (**a**–**c**) Genomic instability at the *Igk* locus in *v-abl* pro-B cell lines. (**a**) Schematic representation of the *Igk* locus, with positions of the BACs used for generation of DNA FISH probes indicated. (**b**) Representative metaphase from *Rag2*^*c/c*^ *XLF*^*−/−*^ *p53*^*−/*−^ *v-abl* pro-B cell lines using the *Igk* *C* BAC probe (red) combined with *Igk V* BAC probe (green) and chromosome 6 paint (white). Yellow arrowheads point to broken or translocated chromosome 6. (**c**) Percentage of aberrant metaphases from *v-abl* pro-B cell lines of the indicated genotype harbouring chromosomes breaks (white) or translocations (black) involving the *Igk* locus. Histograms represent means+s.e.m. of two to three independent cell lines (see also Supplementary Table 3). ***P<0.001. (**d**–**f**) Genomic instability at the *Igh* locus in primary pro-B cells. (**d**) Schematic representation of the *Igh* locus, with positions of the BACs used for generation of DNA FISH probes indicated. (**e**) Representative metaphase from *Rag2*^*c/c*^ *XLF*^*−/−*^ *p53*^*−/−*^ pro-B cells using the *Igh C* BAC probe (red) combined with *Igh V* BAC probe (green) and chromosome 12 paint (white). Yellow arrowheads point to broken or translocated chromosome 12. (**f**) Percentage of aberrant metaphases from pro-B cells of the indicated genotype harbouring chromosomes breaks (white) or translocations (black) involving the *Igh* locus. Histograms represent means±s.e.m. of at least three independent experiments (See also Supplementary Table 4). ***P<0.001.

We next hypothesized that unrepaired DNA breaks in G1 arrested *Rag2*^*c/c*^ *XLF*^*−/−*^ pro-B cells might lead to chromosomal instability upon re-entry into the cell cycle. To test our hypothesis, we performed DNA-FISH on chromosome spreads prepared from untreated cycling cells and G1-arrested cells released back into cell cycle by removal of ABLki. Using probes centromeric (*Igk V*) and telomeric (*Igk C*) to the *Igk* locus together with specific paint for chromosome 6, we quantified the frequency of *Igk* chromosome breaks and translocations (Fig. 6a–c, Supplementary Table 3). Since DNA breaks induce strong p53-dependent G1/S checkpoint and apoptotic signalling, we generated additional *v-abl* pro-B cell lines derived from *p53*^*−/−*^, *Rag2*^*c/c*^ *p53*^*−/−*^, *XLF*^*−/−*^ *p53*^*−/−*^ and *Rag2*^*c/c*^ *XLF*^*−/−*^ *p53*^*−/−*^ mice. Interestingly, metaphases prepared from released *Rag2*^*c/c*^ *XLF*^*−/−*^ *p53*^*−/−*^ pro-B cells harboured a statistically significant increase in genomic instability, most notably translocations (17.7%, *n*=724), as compared with *p53*^*−/−*^ (6%, *n*=745), *Rag2*^*c/c*^ *p53*^*−/−*^ (8%, *n*=250) and *XLF*^*−/−*^ *p53*^*−/−*^ (5.7%, *n*=403) pro-B cells (Fig. 6c and Supplementary Table 3). To determine whether chromosome breaks and translocations were detectable in primary B cell progenitors, we cultured bone marrow CD19^+^IgM^−^ cells and examined metaphases using probes centromeric (*Igh C*) and telomeric (*Igh V*) to the *Igh* locus plus specific paint for chromosome 12 (Fig. 6d–f and Supplementary Table 4). We found very low levels of *Igh* breaks and translocations in p53 proficient pro-B cells (Supplementary Table 4). In contrast, we detected a significant increase in these lesions in *Rag2*^*c/c*^ *XLF*^*−/−*^ *p53*^*−/−*^ pro-B cells (10.3%, *n*=943) when compared with *p53*^*−/−*^ (1.7%, *n*=421), *Rag2*^*c/c*^ *p53*^*−/−*^ (3.5%, *n*=883) and *XLF*^*−/−*^ *p53*^*−/−*^ (4.2%, *n*=910) pro-B cells (Fig. 6f and Supplementary Table 4). We conclude that *Rag2*^*c/c*^ *XLF*^*−/−*^ primary pro-B cells, like *v-abl* pro-B cell lines, harbour high levels of genomic aberrations at the rearranging *Ig* loci that persist in the absence of p53-dependent cell cycle/apoptotic checkpoint. These results reveal unique redundant functions between RAG2 and XLF in suppressing genomic instability during antigen receptor gene assembly.

### *Rag2* ^*c/c*^ *XLF* ^*−/−*^ *p53* ^*−/−*^ mice develop pro-B cell lymphomas

**Figure 7:**
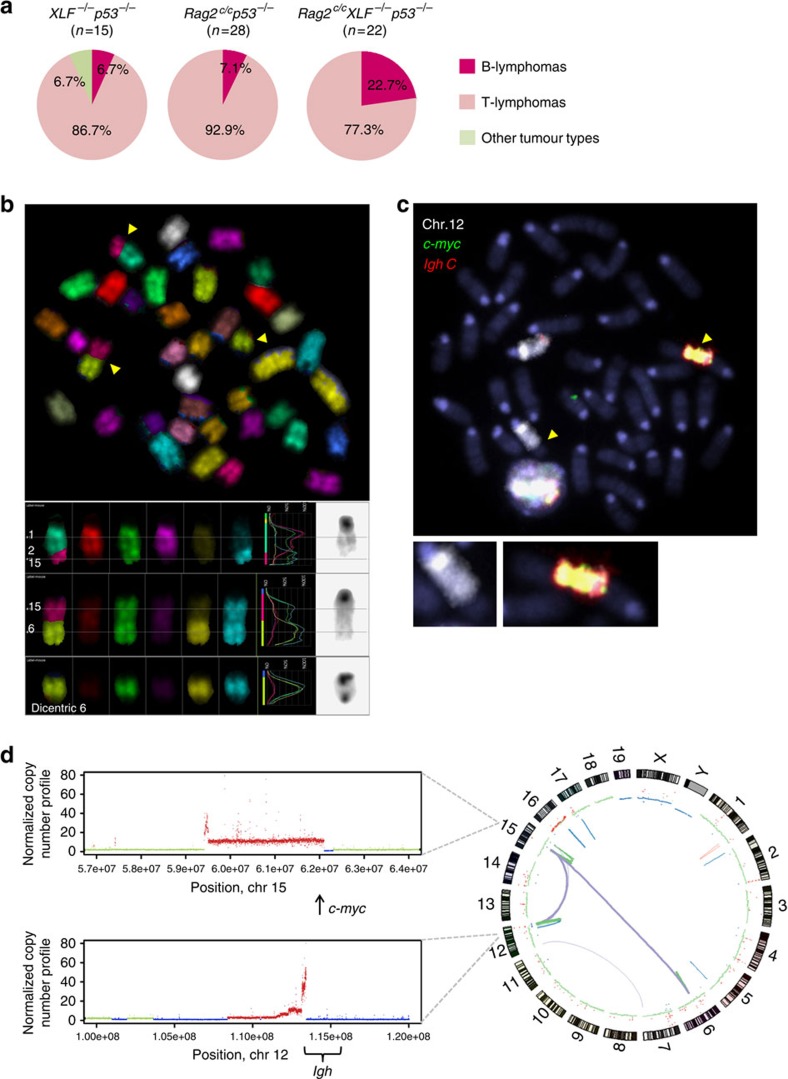
*Rag2*^*c/c*^ *XLF*^*−/−*^ *p53*^*−/−*^ mice develop pro-B cell lymphomas harbouring *Igh*/*c-myc* translocations associated with gene amplification. (**a**) Tumour spectrum analysis of *XLF*^*−/−*^ *p53*^*−/−*^ (*n*=15), *Rag2*^*c/c*^ *p53*^*−/−*^ (*n*=28) and *Rag2*^*c/c*^ *XLF*^*−/−*^ *p53*^*−/−*^ mice (*n*=22). (**b**) Representative images of spectral karyotyping analysis of 1 *Rag2*^*c/c*^ *XLF*^*−/−*^ *p53*^*−/−*^ B lymphoma (#14189LN). Five B lymphomas were analysed (see also Supplementary Table 6). (**c**) Representative metaphase from *Rag2*^*c/c*^ *XLF*^*−/−*^ *p53*^*−/−*^ B lymphoma (#14189LN) using the *Igh C* BAC probe (red) combined with *c-myc* BAC probe (green) and chromosome 12 paint (white). Experiments were repeated on five different samples. (**d**) Circos plot showing chromosomal rearrangements of B lymphoma #14189LN identified by WGS. Chromosomes are arranged circularly end-to-end with each chromosome's cytobands marked in the outer ring. The inner ring displays copy number data inferred from WGS with blue dots indicating losses, red dots indicating gains and green dots indicating normal copy numbers. Within circles, purple, orange, green and blue lines represent inter-chromosomal rearrangements, duplications, inversions and deletions, respectively. The thickness of the link is proportional to the number of read pairs supporting the structural variant. The left panels depict copy number variations in specific regions of chromosome 12 (bottom) and 15 (upper). A total of 3 *Rag2*^*c/c*^ *XLF*^*−/−*^ *p53*^*−/−*^ B lymphomas were sequenced (see also Supplementary Fig. 11 and Supplementary Data 1).

cNHEJ/p53-deficient mice irremediably develop pro-B cell lymphomas associated with aberrant V(D)J recombination driven clonal translocations and gene amplifications^50^,^51^. In contrast, the majority of *XLF*^*−/−*^ *p53*^*−/−*^ mice succumb to T-cell lymphomas lacking recurrent translocations^20^. Thus, we wondered whether combined RAG2/XLF/p53-triple-deficiency might affect tumour outcomes in these mice. *Rag2*^*c/c*^ *XLF*^*−/−*^ *p53*^*−/−*^ animals survived an average of 11.5 weeks, indistinguishable from *Rag2*^*c/c*^ *p53*^*−/−*^ mice (mean survival=12.4 weeks), but significantly less than *XLF*^*−/−*^ *p53*^*−/−*^ animals (15 weeks) (Supplementary Fig. 10). Seventeen of the twenty-two *Rag2*^*c/c*^ *XLF*^*−/−*^ *p53*^*−/−*^ mice died of T-cell lymphomas harbouring clonal chromosomal translocations involving the *Igh* and *Tcrα/δ* loci, most likely resulting from *Rag2*^*c/c*^ *p53*^*−/−*^ deficiency^30^ (Fig. 7a, Supplementary Fig. 10 and Supplementary Table 5). Strikingly, 5 of the 22 *Rag2*^*c/c*^ *XLF*^*−/−*^ *p53*^*−/−*^ mice succumbed to pro-B cell lymphomas (Fig. 7a and Supplementary Table 6), consistent with the observation that *Rag2*^*c/c*^ *XLF*^*−/−*^ *p53*^*−/−*^ pro-B cells harbour high levels of genomic instability (Fig. 6f). Interestingly, spectral karyotyping and DNA-FISH analysis using *Igh* and *c-myc* specific probes revealed recurrent translocations between chromosomes 12 and 15 associated with co-amplifications of the *Igh* locus and the *c-myc* oncogene in all five B-cell lymphomas (Fig. 7b,c, and Supplementary Table 6), similar to what has been reported in cNHEJ/p53-deficient mice^51^. To further analyse *Rag2*^*c/c*^ *XLF*^*−/−*^ *p53*^*−/−*^ pro-B lymphomas, we whole-genome sequenced three tumours. In all three pro-B cell lymphomas analysed, copy number variation analysis confirmed amplification of the *Igh* locus and *c-myc* (Fig. 7d and Supplementary Fig. 11) and structural variant calling revealed multiple translocations (Fig. 7d, Supplementary Fig. 11 and Supplementary Data 1) among which the most frequent rearrangement fused the *Igh* locus to a sequence downstream of the *c-myc* gene encoding for *pvt1*, a lncRNA (Supplementary Fig. 11b and Supplementary Data 1). Interestingly, most translocation junctions contained short sequence homology indicating possible involvement of alternative NHEJ pathway (Supplementary Fig. 11b and Supplementary Data 1), which is mostly operative in the absence of functional repair by cNHEJ and/or destabilization of the RAG-PCC^3^,^29^,^31^,^32^,^51^. Together, these results indicate that, in the context of XLF deficiency, the RAG complex (more specifically the RAG2 C-terminus) behaves as a genome caretaker that maintains the integrity of the genome by promoting accurate repair of RAG-mediated DNA breaks and suppressing chromosomal rearrangements.

## Discussion

The repair of RAG-induced DSB is known to be fully dependent on core cNHEJ factors (Ku70/80, XRCC4 and Ligase IV) and DNA-PKcs/Artemis in the context of CJ formation^3^,^4^,^5^. XLF is a cNHEJ factor but is not required for V(D)J recombination^19^,^20^. Here, we find that the RAG complex and XLF functionally overlap in the repair of DNA breaks during antigen receptor assembly. Our data provide multiple lines of evidence supporting the conclusion that, in the absence of XLF, the RAG complex plays a direct role in assisting repair of chromosomal RAG DSBs. Contrary to single deficiency due to loss of the C-terminal region of RAG2 (core RAG2) or XLF that do not harbour major defects in V(D)J recombination, we find that combined core RAG2 and XLF deficiency leads to a block in lymphocyte development at the progenitor stage when V(D)J recombination occurs, very reminiscent of the severe combined immunodeficiency phenotype observed in cNHEJ-deficient mice. Utilizing core RAG2/XLF-deficient *v-abl* pro-B cells, we were able to show that (i) these cells, when arrested in G1 to induce RAG expression, accumulate RAG-dependent 53BP1/γH2AX DNA repair foci to similar levels observed in Ku80-deficient cells and display severe impairment in joining RAG DSBs, (ii) rare CJs detected in these cells harbour deletions and microhomologies reminiscent of rare joints seen in core cNHEJ-deficient cells and (iii) in the context of p53 deficiency, these cells and primary pro-B cells accumulate genomic instability in the form of chromosomal breaks and translocations. Lastly, we found that *Rag2*^*c/c*^*XLF*^*−/−*^*p53*^*−/−*^ mice develop pro-B cell lymphomas carrying chromosomal translocations and gene amplification involving the *Igh* and *c-myc* loci, similarly to core cNHEJ/p53 double-deficient mice. These results are consistent with a severe end-joining defect in core RAG2/XLF-deficient cells and demonstrate that core RAG2 B cells require XLF and reciprocally XLF-deficient B cells require RAG to achieve DNA end repair during V(D)J recombination.

A major challenge will be to elucidate the nature of the overlapping functions of XLF and the RAG complex in the repair of RAG-mediated DNA breaks and whether they act in the same general function (for example, end-stabilization/tethering) or in unique pathways (for example, end-stabilization/tethering versus cNHEJ factor recruitment, joining kinetics, end-protection/resection) that are functionally compensatory. Based on our results and previous work, we favour a two-tier synapse model in which both the RAG-PCC and XLF ensure stabilization of DNA ends following RAG1/2 cleavage. ATM and/or ATM-dependent DDR factors would contribute to the RAG-PCC in accord with their role in RAG-DSB stabilization and with their observed functional redundancy with XLF during V(D)J recombination^6^,^8^ (Supplementary Fig. 12—see below).

Consistent with this model, core-RAG2 is known to destabilize the RAG-PCC *in vitro* and to increase the rate of aberrant recombination product formation *in vivo* including HJs and inter-chromosomal translocations involving V(D)J loci^29^,^30^,^31^,^36^,^37^,^39^. XLF forms, with XRCC4, long heterofilaments *in vitro* and *in vivo* potentially capable of maintaining the broken ends together while they are processed for ligation^14^,^15^,^16^,^17^,^18^. Disrupting the interaction between XLF and XRCC4 leads to impaired coding (but not signal) joint formation^52^. Since the RAG proteins bind much more avidly to signal-end pairs than coding-end pairs *in vitro*^2^, this might reflect end-stabilization activities of the XLF-XRCC4 filaments that are more strongly compensated for by signal-end tethering provided by the RAG PCC. Additionally, during inversional V-to-J recombination at the *Igk* locus, similar to *Rag2*^*c/c*^ or *Atm*^*−/−*^ pro-B cells^7^,^30^, *XLF*^*−/−*^ pro-B cells show increased HJ formation, revealing a role for XLF in the stabilization of RAG-induced chromosomal DSBs. Notably, Southern blotting analysis revealed that, in addition to rearranged products, low levels of unrepaired coding ends are detectable at the *Igk* locus in *XLF*^*−/−*^ pro-B cells. The presence of these unrepaired coding ends is consistent with a role for XLF in stabilizing RAG-DSBs or with a role in general DSB joining^18^,^53^,^54^. Therefore, in addition to DNA end destabilization, a mild decrease in joining kinetics and/or efficiency caused by XLF deficiency alone^18^,^21^,^53^,^54^ might also contribute to the observed end-joining defect in *Rag2*^*c/c*^*XLF*^*−/−*^ cells.

Absence of a double safe-lock provided by RAG and ATM-DDR on one side and XLF/XRCC4 on the other side might ultimately cause the collapse of PCCs (Supplementary Fig. 12), leading to release/vulnerability of DNA ends and an absence of *cis*-rearrangement products. This is consistent with the observation of severely impaired joining of RAG-generated DSBs in cells that are doubly deficient for XLF and either ATM, 53BP1 or H2AX (ref. 8) as well as in core RAG2/XLF-deficient cells. Notably, while un-joined DNA ends remain intact in G1-arrested XLF/ATM double-deficient pro-B cells, they are highly resected in XLF/H2AX or XLF/53BP1 double-deficient cells because of the DNA end resection activity of CtIP^49^, and possibly KAP-1 (ref. 55). During V(D)J recombination, ATM has a dual impact on DNA end fate, both protecting these ends from release/resection possibly via their stabilization within PCCs and promoting their resection by activating CtIP. Therefore, RAG-cleaved DNA ends might be released/exposed in all cases where the two locks (ATM-DDR/RAG PCC and XLF) are missing but only subjected to resection in the presence of a functional ATM kinase. Correspondingly, inhibition of the ATM kinase activity in XLF/H2AX or XLF/53BP1 double-deficient pro-B cells restores accumulation of intact unrepaired DNA ends^8^,^21^. Similarly, in *Rag2*^*c/c*^*XLF*^*−/−*^ pro-B cells, intact unrepaired DNA ends were rescued by treatment of cells with an ATM kinase inhibitor.

It is tantalizing to speculate that the RAG proteins and the ATM-DDR participate in the same pathway that is functionally redundant with XLF. This is supported by our findings showing that (1) *Rag2*^*c/c*^*Atm*^*−/−*^ mice display relatively normal B and T lymphocyte development, (2) V(D)J recombination is not abolished in *Rag2*^*c/c*^*Atm*^*−/−*^ *v-abl* pro-B cells, (3) *Rag2*^*c/c*^*Atm*^*−/−*^ developing B and T cells and *v-abl* pro-B cells harbour similar levels of genomic instability at V(D)J loci as compared with ATM-deficient cells and (4) *Rag2*^*c/c*^*Atm*^*−/−*^ mice develop aggressive T-cell lymphomas-bearing complex chromosomal translocations, amplifications and deletions involving the *Tcrα/δ* and *Igh* loci, similarly to lymphomas that develop in *Atm*^*−/−*^ and *Rag2*^*c/c*^*p53*^*−/−*^ mice. We do not know yet whether the RAG and ATM-DDR end-stabilization/protection function(s) requires direct biological interactions between the two complexes. 53BP1/γH2AX foci accumulate at similar levels in core RAG2 pro-B cells as compared with WT pro-B cells and at higher levels in core RAG2/XLF-deficient cells, indicating that the RAG2 C-terminus is not required for initial sensing of cleaved DNA ends (that is, the phosphorylation of H2AX and accumulation of 53BP1 at RAG DSBs) by the ATM-DDR machinery. ATM and/or DNA-PKcs mediated phosphorylation of RAG1 and RAG2 proteins is not required for recombination of chromosomally integrated substrates in WT *v-abl* pro-B cells^56^; but whether such activity could contribute to the impact of ATM/RAG deficiency on V(D)J recombination in the absence of XLF remains to be tested^8^. In response to genotoxic damage induced by irradiation, ATM phosphorylates hundreds of proteins active in different aspects of the DDR^57^. In that regards, ATM-DDR is likely to involve more than one pathway that could functionally compensate for XLF deficiency. In the context of V(D)J recombination, we suggest that the function of one of these pathways is to stabilize/protect DNA ends and requires the RAG proteins.

Our work raises the possibility that normal physiological antigen receptor assembly may have evolved to optimize ability of the RAG endonuclease to assist repair of generated DNA breaks within post-cleavage complexes, providing an additional layer of protection against aberrant joining, genomic instability and cell transformation. As the RAG proteins are only expressed in developing lymphocytes, such a role would necessarily overlap with the ubiquitous DNA damage response and/or cNHEJ machineries—here, we identify XLF as one component with which the RAG complex may have functionally co-evolved.

## Methods

### Mice

We obtained WT (Taconic), *p53*^*+/−*^ (Jackson Laboratory^58^), *Atm*^*+/−*^ (Jackson Laboratory^41^), *Rag2*^*−/−*^ (Taconic^48^), *Ku80*^*+/−*^ (Jackson Laboratory^59^), *XLF*^*−/−19*^, *Rag2*^*c/c30,34*^ and *Rag2*^*c/c*^ *p53*^*+/−30*^ mice for this study. *XLF*^*−/−*^ mice were bred with p53-deficient mice to generate doubly deficient mice. *Rag2*^*c/c*^ mice were bred with ATM-deficient mice to generate doubly deficient mice. *Rag2*^*c/c*^ were bred to *XLF*^*−/−*^ mice to generate doubly deficient mice*. Rag2*^*c/c*^ *p53*^*+/−*^ mice were bred to *Rag2*^*c/c*^ *XLF*^*−/−*^ deficient mice to generate triply deficient animals. Genotyping of these mutants was performed by PCR of tail DNA as described in the relevant references. All animal experiments were performed in accordance with the guidelines of the institutional animal care committee of Institut Pasteur/CEEA Ile-de-France-Paris1 under the protocol number 2012-0036.

### Lymphocyte development

Lymphocyte development was analyzed in the thymus, bone marrow and spleen from 4 to 8-week-old mice. All single-cell suspensions were treated with Fc-blocking antibody (CD16–32, 1:200 dilution) before cell surface staining, which was performed in phosphate-buffered saline (PBS) with 2% fetal bovine serum for 30 min at 4 °C. Bone marrow B lineage cell populations were identified based on the expression of the following markers: pro-B (B220^lo^ CD43^+^ CD19^+^ IgM^−^), pre-B (B220^lo^ CD43^−^ CD19^+^ IgM^−^), immature B cells (B220^lo^ CD43^−^ CD19^+^ IgM^+^), recirculating B cells (B220^hi^ CD43^−^ CD19^+^ IgM^+^). T lineage cell populations from the thymus were identified based on the expression of the following markers: double-negative (DN) cells (CD4^−^CD8^−^), DN1 (CD4^−^CD8^−^CD44^+^CD25^−^), DN2 (CD4^−^CD8^−^CD44^+^CD25^+^), DN3 (CD4^−^CD8^−^CD44^−^CD25^+^), DN4 (CD4^−^CD8^−^CD44^−^CD25^−^), double-positive (DP) cells (CD4^+^CD8^+^) and single-positive (SP) cells (CD4^+^CD8^−^ and CD4^−^CD8^+^). Lymphocytes from the spleen were identified based on the expression of the following markers: B cells (CD19^+^IgM^+^) and T cells (CD3^+^TCRβ^+^). The following antibodies were used for cell surface staining: anti-B220 (RA3–6B2, 1:200 dilution), anti-CD43 (S7, 1:150 dilution), CD19 (1D3, 1:200 dilution), anti-IgM (R6–60.2, 1:150 dilution), anti-IgD (11–26c.2a, 1:200 dilution), anti-c-Kit (2B8, 1:300 dilution), anti-CD4 (RM4–5, 1:200 dilution), anti-CD8a (53-6.7, 1:200 dilution), anti-CD3e (145-2C11, 1:200 dilution), anti-CD44 (IM7, 1:200 dilution), anti-CD25 (PC61, 1:200 dilution) and anti-TCRβ (H57–597, 1:200 dilution). All antibodies were purchased from BD Biosciences except anti-TCRβ, which was from eBiosciences. Flow cytometry was performed on a FACS Canto II (BD Bioscience) and data were analysed using FlowJo (TreeStar).

### *In vitro* culture of B-cell progenitors

Freshly isolated bone marrow cells from 2–4 mice (4–8 weeks old) were pooled and depleted of IgM^+^ cells by magnetic separation using IgM microbeads (Miltenyi). IgM^−^ cells were then incubated with CD19 microbeads (Miltenyi) and enriched for CD19^+^ cells. Bone marrow CD19^+^IgM^−^ cells were cultured for 3–4 days in complete Opti-MEM supplemented with IL-7 (50 ng ml^−1^), SCF (50 ng ml^−1^) and Flt3L (50 ng ml^−1^) to stimulate proliferation of pro-B cells before metaphase preparation.

### Generation of *v-abl* transformed pro-B cell lines

Total bone marrow from 3–5-week-old mice of each genotype was cultured and infected with a retrovirus encoding *v-abl* kinase to generate immortalized pro-B cell lines^60^. *v-abl* transformed pro-B cell lines were then transduced with pMSCV-*Bcl2*-puro retrovirus^61^ to protect them from *v-abl* kinase inhibitor-induced cell death (Supplementary Fig. 5a).

### V(D)J recombination assays

The pMX-INV or pMX-DEL^CJ^ substrate was introduced in pro-B cell lines through retroviral infection and cells that had integrated the recombination substrate were enriched based on hCD4 expression^7^,^34^. For V(D)J recombination assay, *v-abl* transformed *Bcl2*/pMX-INV infected pro-B cells (10^6^ per ml) were treated with 3 μM of the *v-abl* kinase inhibitor STI571 (Novartis) or 0.3 μM of the STI571-analogous *v-abl* kinase inhibitor PD180970 (Sigma) and assayed for rearrangement by FACS analysis of GFP expression or Southern blotting at 0, 72 or 96 h. In some experiments, the ATM kinase inhibitor KU55933 was added at 15 μM together with PD180970 or STI571 (named ABLki). For FACS analysis, V(D)J recombination efficiency was scored as the percentage of GFP positive cells among hCD4-positive cells (human CD4-PE, Miltenyi, 1:20 dilution). For ABLki release experiments, cells were collected, washed and cultured without ABLki for 3–4 days before metaphases preparation.

### Southern blot

30 μg of gDNA from untreated, ABLki-treated and ABLki/ATMki-treated pro-B cell lines were digested overnight with EcoRV for pMX-DEL^CJ^, EcoRV or EcoRV/NcoI for pMX-INV and SacI/EcoRI for endogenous *Igk* locus analysis. Digested gDNA samples were run overnight on an agarose gel, denatured by incubating the gel with 0.5 M NaOH/0.6 M NaCl for 1 h, and then transferred overnight on a Zeta-Probe GT nylon membrane (Biorad). DNA was cross-linked on the membrane using a UV Cross-linker CL-508 (Uvitec Cambridge). Blots were incubated at 42 °C in pre-hybridization buffer for at least 1 h and then overnight in hybridization buffer containing a 32^P^-CTP labelled probe: the C4 probe for pMX-INV and pMX-DEL^CJ 7,21^ and the JkIII probe for *Igk* locus^7^,^62^. Blots were washed in 2XSSC/0.1% SDS at 65 °C and exposed to a Storage Phosphor Screen (GE Healthcare) for 2–5 days. The screen was then scanned using a Storm 860 PhosporImager (Molecular Dynamics).

### PCR analysis of V(D)J recombination products

pMX-DEL^CJ^ CJs were amplified using pC (5′-GCACGAAGTCTTGAGACCT-3′) and IRES-REV5 (5′-CTCGACTAAACACATGTAAAGC-3′) oligonucleotides as previously described^7^,^49^. Oligonucleotide IR4 (5′-CCCTTGTTGAATACGCTTG-3′) was used as a probe for pMX-DEL^CJ^ CJs. Il2 gene was amplified using IMR42 (5′-CTAGGCCACAGAATTGAAAGATCT-3′) and IMR43 (5′-GTAGGTGGAAATTCTAGCATGATGC-3′) primers and was used as loading control. pMX-DEL^CJ^ CJs were cloned using TOPO TA Cloning kit (Life Technologies) following manufacturer's instructions and analyzed by Sanger Sequencing using T3 (5′-AATTAACCCTCACTAAAGGGA-3′) and T7 (5′-TAATACGACTCACTATAGG-3′) primers. Endogenous *Vκ*_*6-23*_*/Jk*_*1*_ CJs and HJs were amplified as previously described^7^. A total of 500 ng of genomic DNA was amplified using pkJa (5′-GGAGAGTGCCAGAATCTGGTTTCAG-3′) and pk6a (5′-TGCATGTCAGAGGGCACAACTG-3′) primers for HJ and pkJa2 (5′-GCCACAGACATAGACAACGGAA-3′) and pk6d (5′-GAAATACATCAGACCAGCATGG-3′) primers for CJ. Serial 4-fold dilutions of this reaction were amplified using pkJa and pk6b (5′-CTACCAAACTTTGCAACACACAGGC-3′) primers for HJ and pkJa2 and pk6c (5′-GTTGCTGTGGTTGTCTGGTG-3′) primers for CJ.

### Metaphase preparation

Metaphases were prepared using standard procedures^30^. Briefly, cells were incubated with colcemid (0.03 μg ml^−1^, Life technologies, KaryoMAX Colcemid Solution) for 3–4 h at 37 °C. Then, cells were collected and incubated in 0.075 M KCl for 10 min at 37 °C, fixed in fixative solution (75% methanol/25% acetic acid) and washed three times in the fixative. Cell suspension was dropped onto humid slides and air-dried for further analysis.

### DNA-FISH probes

BAC probes RP24–134G24 (5' *Igh* C), RP24–386J17 (3' *Igh* V), RP24–243E11 (5' *Igk* V), RP23–341D5 (3' *Igk* C) and RP24–307D14 (*c-myc*) were directly labelled by nick translation with ChromaTide Alexa Fluor 488- or 594-5-dUTP (Life technologies), or Cy3-dUTP (GE Healthcare), as previously described^63^. Nick translation reaction was optimized to get a DNA smear between 100 and 600 bp. Labelled BAC probes were purified using Illustra MicroSpin G-50 Columns (GE Healthcare). A total of 600 ng of each locus-specific BAC probes were pooled and precipitated with mouse Cot1 DNA and Salmon Sperm DNA (Life Technologies). Probes were then re-suspended in hybridization buffer (10% dextran sulphate, 5 × Denharts solution, 50% formamide), denatured for 5 min at 95 °C and pre-annealed for 30–45 min at 37 °C. XCyting Mouse Chromosome 6, 12 or 14 (Red or Orange) paints from MetaSystems were denatured for 5 min at 95 °C and mixed with BAC probes just before hybridization.

### DNA FISH on metaphase spreads

Slides were treated with RNase A for 40 min, dehydrated in 70, 90 and 100% ethanol for 3 min each, denatured in 70% formamide/2 × SSC for 3 min at 77 °C, dehydrated again in cold ethanol series, and hybridized with probes o/n at 37 °C in a humid chamber. The next day, slides were washed three times in 50% formamide/0.5 × SSC for 5 min each at 37 °C and twice in 0.5 × SSC for 10 min each at 37 °C. Finally, slides were mounted in ProLong Gold (Life Technologies) containing 49,6-diamidino-2-phenylindole (DAPI) to counterstain total DNA. Metaphases were imaged using a ZEISS AxioImager.Z2 microscope and the Metafer automated capture system (MetaSystems), and counted manually.

### Immuno-DNA FISH on interphase nuclei

After 3d treatment with PD180970, pro-B cell lines were adhered to poly-L lysine-coated coverslips and stained as previously described^63^ with minor changes. Cells were fixed with 4% paraformaldehyde/PBS for 10 min at room temperature (RT) and permeabilized for 5 min with 0.4% Triton/PBS on ice. Immunofluorescence was performed after 30 min blocking in 3% bovine serum albumin/PBS, with a primary antibody against 53BP1 (NB100–304SS, 1:400 dilution, Novus Biologicals) and a secondary goat-anti-rabbit antibody (Alexa Fluor 594, 1:800 dilution; Life Technologies) in blocking solution, each for 1 h at RT. For co-localization experiments with γH2AX, FITC-labelled antibody against phosphorylated serine-139 of H2AX (γ-H2AX-FITC, clone JBW301, 1:400 dilution, Millipore) was added at the secondary staining step. Cells were rinsed three times in 0.5% bovine serum albumin/PBS, and post-fixed in 4% paraformaldehyde/PBS for 10 min at RT. Cells for both DNA FISH and immuno-FISH were then incubated with 0.01 mg ml^−1^ RNaseA for at least 1 h at 37 °C and permeabilized in 0.7% Triton X-100/0.1 M HCl for 10 min on ice. Cells were then denatured with 2 N HCl for 30 min at RT, rinsed three times in iced-cold PBS and hybridized overnight at 37 °C with 0.6–1 μg of *Igk*C probe (coverslips were sealed onto slides with rubber cement). The next day, cells were rinsed in 2 × SSC at 37 °C, then in 2 × SSC at RT and finally in 1 × SSC at RT, for 45 min each. Finally slides were mounted in DAPI/Antifade reagent (MetaSystems). Cells were imaged in 3D (9 Z stacks of 0.5 μm) using a Zeiss AxioImager Z2 microscope and the Metacyte automated capture system (Metasystems). FISH signals were counted using a custom Metacyte classifier. >10,000 nuclei were counted for each genotype (see Supplementary Table 1 for details).

### Characterization of tumour cells

Lymphoid tumours were analysed by flow cytometry with antibodies against surface B-cell (anti-B220 (RA3–6B2, 1:200 dilution), anti-CD43 (S7, 1:150 dilution), CD19 (1D3, 1:200 dilution), anti-IgM (R6–60.2, 1:150 dilution), anti-IgD (11–26c.2a, 1:200 dilution), anti-c-Kit (2B8, 1:300 dilution)) and T-cell markers (anti-CD4 (RM4–5, 1:200 dilution), anti-CD8a (53-6.7, 1:200 dilution), anti-CD3e (145-2C11, 1:200 dilution), anti-CD44 (IM7, 1:200 dilution), anti-CD25 (PC61, 1:200 dilution), and anti-TCRβ (H57-597, 1:200 dilution)). Flow cytometry was performed on a FACS Canto II (BD Bioscience) and data were analysed using FlowJo (TreeStar). Tumour cells were cultured for 2 h in complete RPMI medium before metaphases preparation. Metaphases spreads were stained with 21 × Mouse, Multicolour Painting mFISH Probe Kit (MetaSystems), which was prepared following supplier's instructions. Slides were mounted in 90% DAPI/Antifade reagent (MetaSystems)/10% ProLong Gold (Life Technologies). Metaphases were imaged using a ZEISS AxioImager.Z2 microscope and the Metafer automated capture system (MetaSystems). Karyotyping was performed using Isis software (MetaSystems).

### Statistics

For lymphocyte development, V(D)J recombination and 53BP1 focus formation assays on *v-abl* pro-B cells, statistical analyses were performed using the non-parametric Mann–Whitney *U*-test. For DNA FISH experiments, Fisher exact tests were used to determine statistical significance (see Supplementary Tables 3 and 4 for details). Prism GraphPad Software was utilized for generating Kaplan–Meier mouse tumour-free survival plots and statistical analysis of survival and tumour onset (log-rank test). In all statistical tests, *P* values<0.05 were taken to be significant (0.01≤*P*<0.05 * significant; 0.001≤*P*<0.01 ** very significant; *P*<0.001 *** highly significant).

### Whole-genome sequencing

The 3 B-cell lymphomas that were sequenced originate from the lymph nodes of three different *Rag2*^*c/c*^ *XLF*^*−/−*^ *p53*^*−/−*^ mice: 13111 (female, 9.3 week old), 14189 (male, 9.1 week old), 14821 (male, 11.1 week old).

Genomic DNA was prepared from single-cell suspension of tumour lymph nodes using Wizard Genomic DNA purification Kit (Promega). Whole-genome library was generated according to the Paired-End Sample Preparation kit (Illumina) protocol. A total of 100 nucleotides paired-end sequencing was performed on an Illumina Genome Analyzer HiSeq 2000 instrument.

Short read sequences were mapped to the reference mouse genome (mm10, Ensembl74) using the Burrows–Wheeler Aligner version 0.7.4 (BWA^64^) algorithm with default parameters except the option -q 25 for read trimming. Duplicate reads were removed using the function MarkDuplicates from Picard tools and a filtering for uniquely mapped reads was performed. We obtained 385 millions uniquely mapped paired-end reads corresponding to 28 × coverage of the mouse genome.

Structural variations (SVs) were predicted by SVDetect^65^ version r0.8b, which uses discordant mapped read pairs provided by the aligner to indicate potential genomic variations from the reference. Mean insert size and standard deviation were computed using the function CollectInsertSizeMetrics from Picard tools. Discordant read pairs with low BWA mapping quality scores (the threshold was set to 23) were removed. SVDetect links2compare function was used for comparison of the tumour and control samples, and we disabled the option for comparing only links sharing the same SV type. We removed SVs identified in the genomes of control samples with at least one read pair. We used four genomes representative of our in-laboratory mouse strains for controls. We removed SVs supported with <4 read pairs. We also removed SVs found in highly repeated regions, by requiring <80 or 50% overlap with RepeatMasker and simple repeats tracks respectively (UCSC). The overlap with repeats was computed with BEDTools. Circos^66^ plot was used to visualize chromosomal rearrangements and copy number variations. We used Socrates^67^ and Delly^68^ version 0.5.6 to identify breakpoints of SVs with single-nucleotide resolution and Socrates to predict micro-homologies and untemplated sequences at breakpoints.

Copy number variants were detected with Control-FREEC algorithm, which uses coverage depth differences to identify amplified or deleted regions^69^. Read count is calculated in sliding windows (window size was set to 50,000 bp) and control sample was used to normalize read count in the tumour sample. Copy number profiles per chromosome were visualized using R.

## Additional information

**Accession codes:** The whole genome sequencing data from the lymphomas has been deposited at the Sequencing Read Archive under accession code SRP067544.

**How to cite this article:** Lescale, C. *et al.* RAG2 and XLF/Cernunnos interplay reveals a novel role for the RAG complex in DNA repair. *Nat. Commun.* 7:10529 doi: 10.1038/ncomms10529 (2016).

## Supplementary Material

Supplementary Information Supplementary Figures 1-12 and Supplementary Tables 1-6

Supplementary Data 1 Structural variants identified by whole genome sequencing of 3 Rag2c/c XLF-/- p53-/- B cell lymphomas
