# Supplementary material for: RAG2 and XLF/Cernunnos interplay reveals a novel role for the RAG complex in DNA repair
Source: Nat Commun. 2016 Feb 2;7:10529. doi: 10.1038/ncomms10529 (PMC4740868; doi:10.1038/ncomms10529)
Supplement: Supplementary Information — Supplementary Figures 1-12 and Supplementary Tables 1-6 [file ncomms10529-s1.pdf]

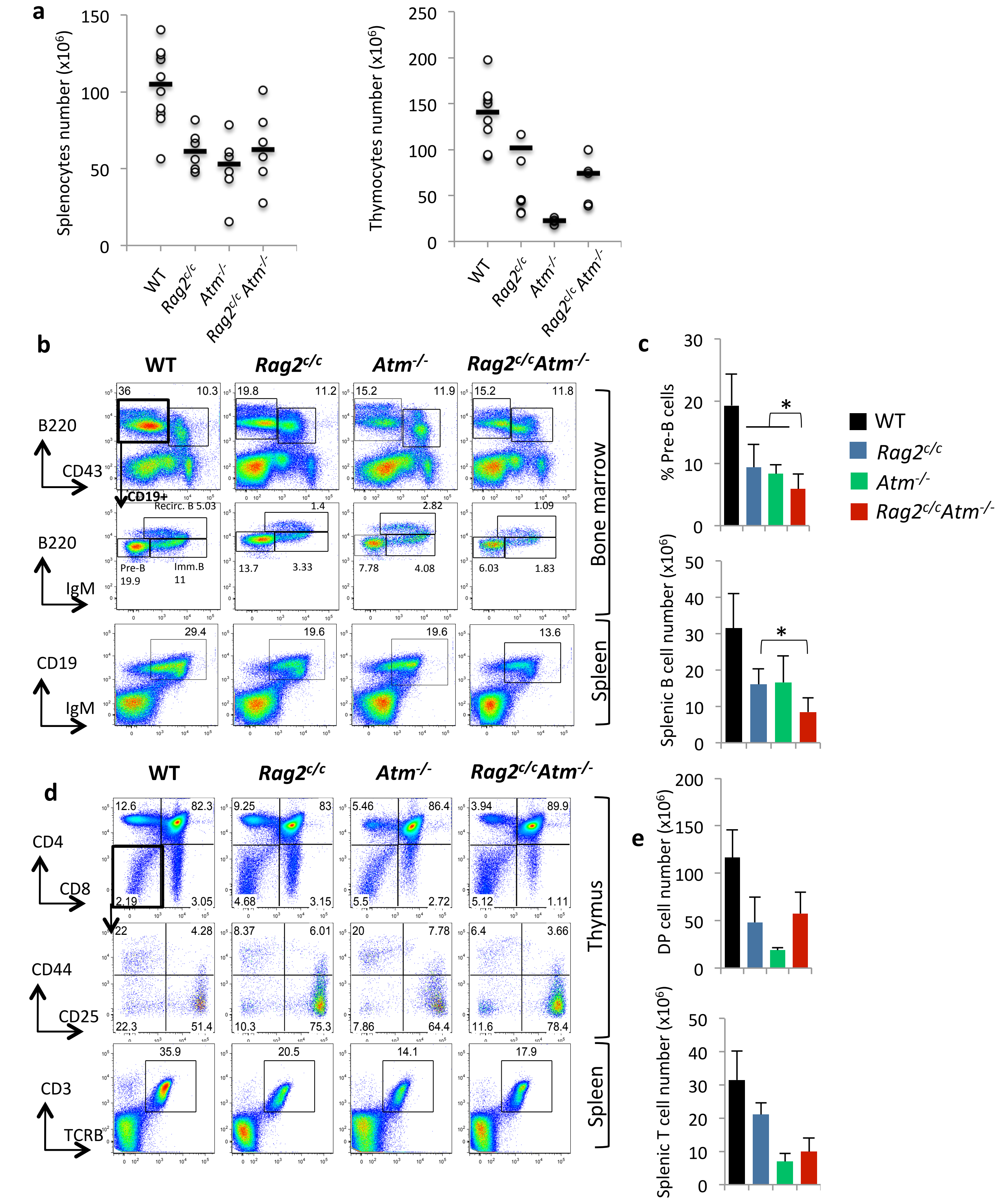

Supplementary Figure 1. Normal lymphocyte development in *Rag2*$^{c/c}$ *Atm*$^{-/-}$ animals.
Lymphocyte development in WT, *Rag2*$^{c/c}$, *Atm*$^{-/-}$, and *Rag2*$^{c/c}$ *Atm*$^{-/-}$ mice. **a**. Total splenocytes (left panel) and thymocytes (right panel) numbers. Each dot represents one mouse and bars indicate the median. WT, *Rag2*$^{c/c}$ and *Rag2*$^{c/c}$ *Atm*$^{-/-}$ mice are on mixed B6/129 backgrounds, whereas *Atm*$^{-/-}$ mice are on pure 129 background. **b.c.** Analysis of B cell development. **b**. Representative FACS analysis of bone marrow and spleen using B cell markers. Numbers on plots represent percentages of total cells. **c**. Percentage of bone marrow CD43^{-}B220^{lo}CD19^{+}IgM^{-} pre-B cells (upper panel) and total number of splenic CD19^{+}IgM^{+} B cells (bottom panel). **d.e**. Analysis of T cell development.
d. Representative FACS analysis of thymus and spleen using T cell markers. **e**. Total number of thymic DP cells (upper panel) and splenic CD3^{+}TCRβ^{+} T cells (bottom panel). Histograms represent means ± SD from at least 5 mice of each genotype. * $0.01 \leq p < 0.05$.

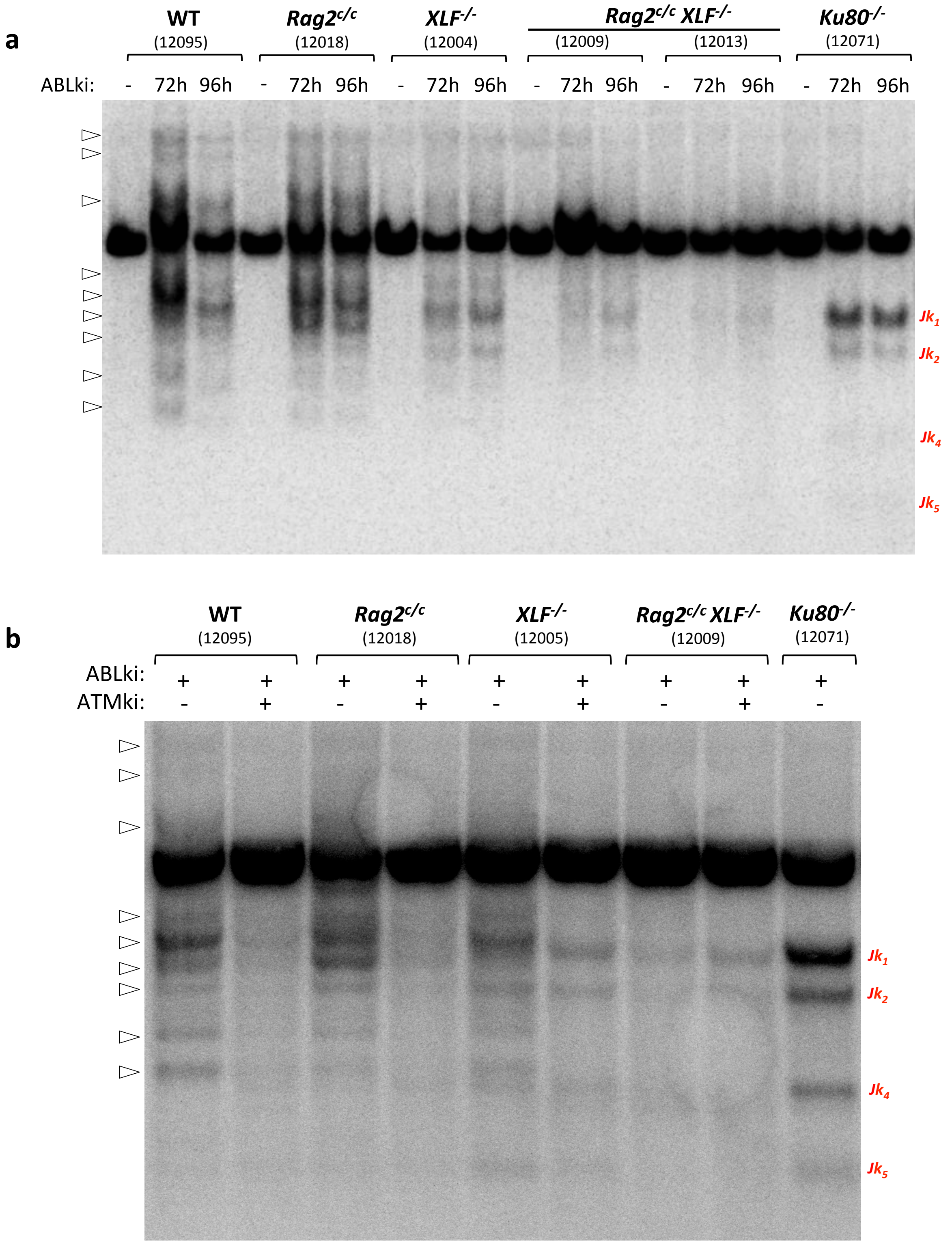

Supplementary Figure 2. Analysis of endogenous *Igk* rearrangements in *v-abl* pro-B cell lines.
a. Southern blot with JKIII probe of EcoRI/SacI digested DNA from *v-abl* pro-B cell lines untreated and treated 72 or 96 hours with ABLki. **b.** Southern blot with JKIII probe of EcoRI/SacI digested DNA from *v-abl* pro-B cell lines treated for 72h with ABLki with or without ATMki. Arrows indicate normal rearrangements. $Jk_{1\text{-}5}$ CEs are indicated in red.

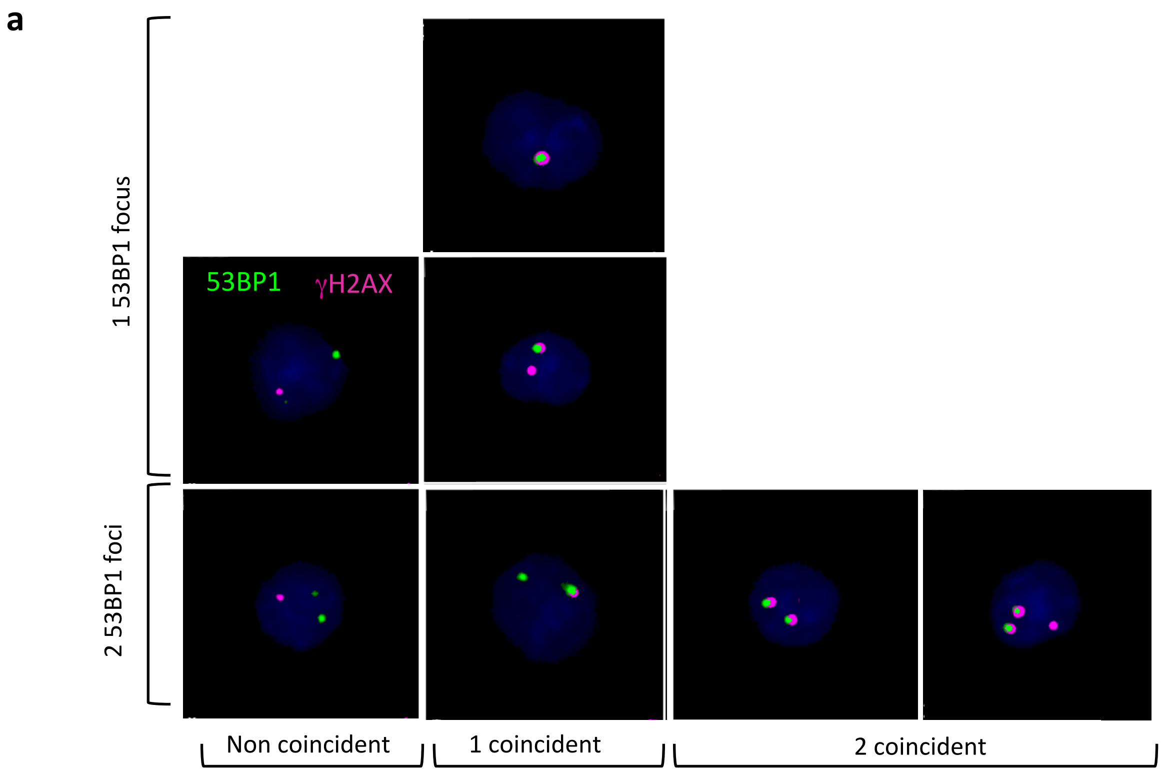

b

Cell class	Coincidence	WT (12095)	*Rag2*$^{c/c}$ *XLF*$^{-/-}$ (12009)	*Rag*$^{-/-}$ (129M)	*Rag*$^{-/-}$ (129F)
Cells with 1 53BP1 focus	non coincident with γH2AX	741 (47.1%)	683 (28.1%)	374 (74.2%)	294 (77.4%)
	1 coincident with γH2AX	831 (52.9%)	1748 (71.9%)	130 (25.8%)	86 (22.6%)
	Total	1572	2431	504	380
Cells with 2 53BP1 foci	non coincident with γH2AX	35 (31.5%)	231 (19.0%)	121 (94.6%)	167 (100%)
	1 coincident with γH2AX	41 (36.9%)	433 (35.7%)	7 (5.4%)	0
	2 coincident with γH2AX	35 (31.5%)	549 (45.2%)	0	0
	Total	111	1213	128	167

Supplementary Figure 3. 53BP1 foci co-localize with γH2AX.

a. Representative fluorescent light microscopy images of immuno-3D FISH analysis conducted on ABLki-treated *Rag2*$^{c/c}$ *XLF*$^{-/-}$ *v-abl* pro–B cell lines. Nuclei were stained with 53BP1 (green) and γH2AX (pink). Images show nuclei with non coincident and coincident 53BP1/γH2AX signals. **b**. Table indicates the number (%) of nuclei harboring non coincident and coincident 53BP1/γH2AX signals among total nuclei of each cell class.

a

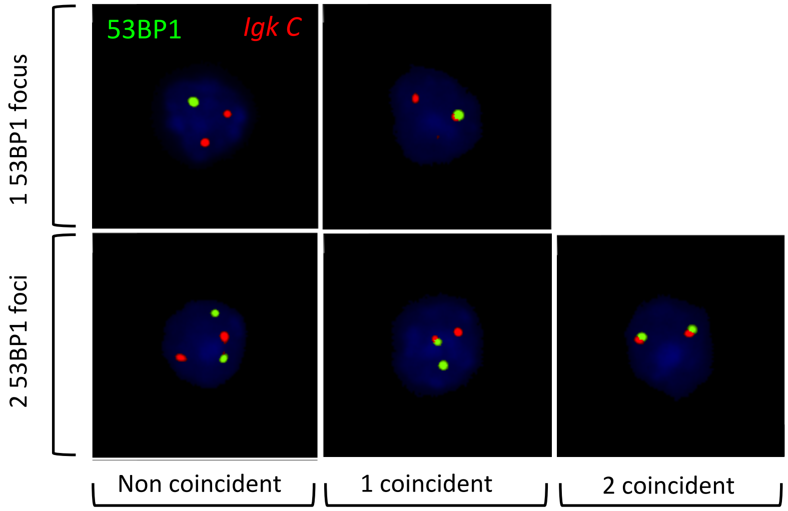

b

Cell class	Coincidence	WT (12095)	*Rag2$^{c/c}$ XLF$^{-/-}$* (12009)	*Rag2$^{c/c}$ XLF$^{-/-}$* (12013)	*Rag$^{-/-}$* (129M)
**Nuclei with 1 53BP1 focus**	**non coincident with *Igk***	389 (38.4%)	318 (20.1%)	291 (19.4%)	172 (68.3%)
	**1 coincident with *Igk***	623 (61.6%)	1266 (79.9%)	1210 (80.6%)	80 (31.7%)
	**Total**	1012	1584	1501	252
**Nuclei with 2 53BP1 foci**	**non coincident with *Igk***	32 (22.9%)	29 (3.1%)	61 (6.6%)	30 (39.0%)
	**1 coincident with *Igk***	83 (59.3%)	255 (26.8%)	316 (34.1%)	47 (61.0%)
	**2 coincident with *Igk***	25 (17.9%)	666 (70.1%)	551 (59.4%)	0
	**Total**	140	950	928	77

Supplementary Figure 4. 53BP1 foci co-localize with the *Igk* locus.
a. Representative fluorescent light microscopy images of immuno-3D FISH analysis conducted on ABLki-treated on *Rag2$^{c/c}$ XLF$^{-/-}$ v-abl* pro–B cell lines. Nuclei were stained with 53BP1 (green) and subsequently hybridized with the centromeric *Igk* BAC probe (*Igk C*, red). Images show nuclei with non-coincident and coincident 53BP1/*Igk* signals. **b.** Table indicates the number (%) of nuclei harboring non-coincident and coincident 53BP1/*Igk* signals among total nuclei of each cell class.

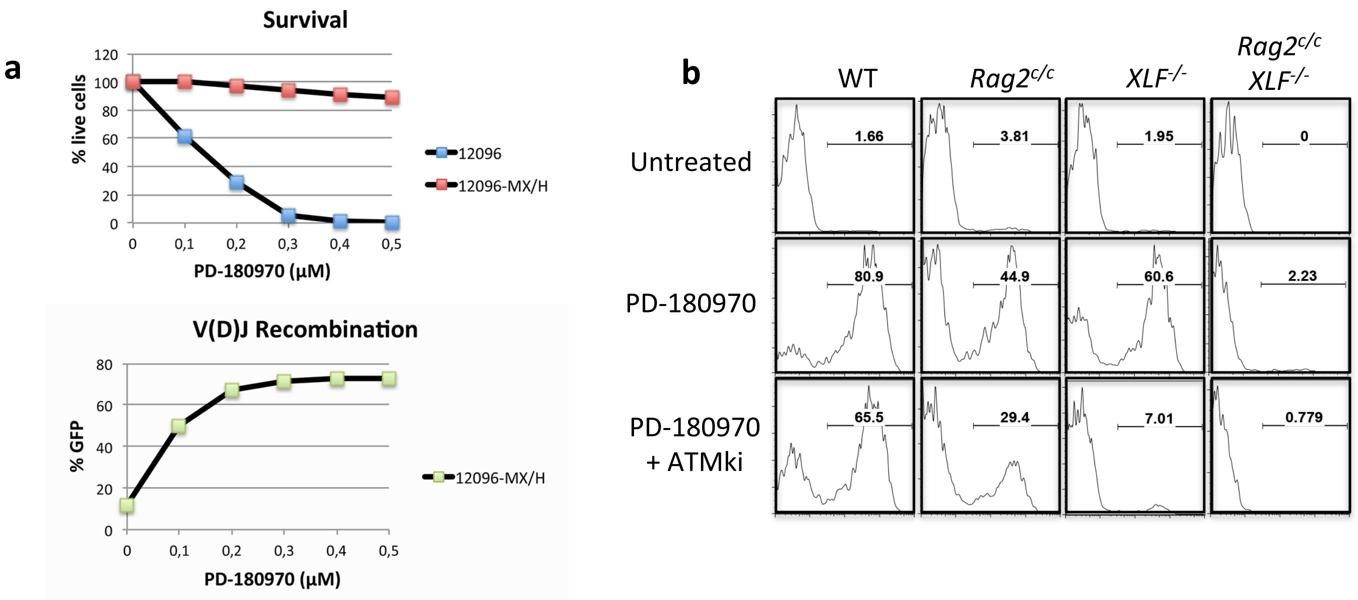

Supplementary Figure 5. V(D)J recombination assays in *v-abl* pro-B cell lines.
a. Determination of optimal dose of PD-180970 to induce V(D)J recombination and preserve cell survival after 96h of treatment in WT *v-abl* pro-B cell lines carrying *bcl-2* and pMX-INV transgenes (12096-MX/H) or not (12096). **b**. Pro-B cells of various genotypes were treated with PD-180970 (0.3µM) for 96h, with or without pre-incubation with KU55933 ATM inhibitor (ATMki, 15µM), and assayed for pMX-INV rearrangement by FACS analysis (numbers indicate percentage of GFP positive cells).

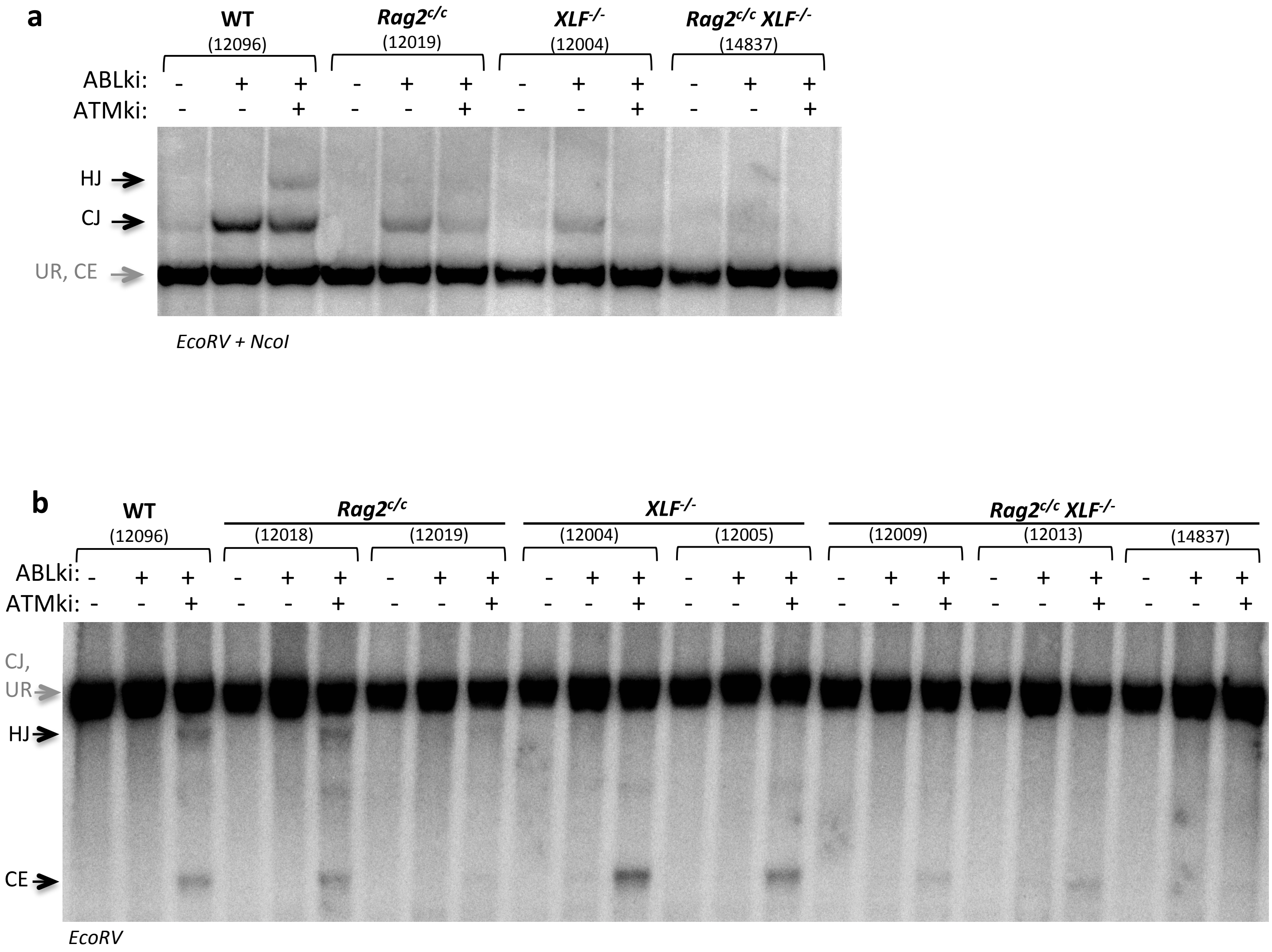

Supplementary Figure 6. Analysis of pMX-INV rearrangements in *v-abl* pro-B cell lines by Southern blotting. The indicated *v-abl* pro-B cell lines containing the pMX-INV substrate were treated for 72h with ABLki with or without ATMki and assayed by Southern blotting; EcoRV/NcoI digest – C4 probe (**a**) and EcoRV digest – C4 probe (**b**).

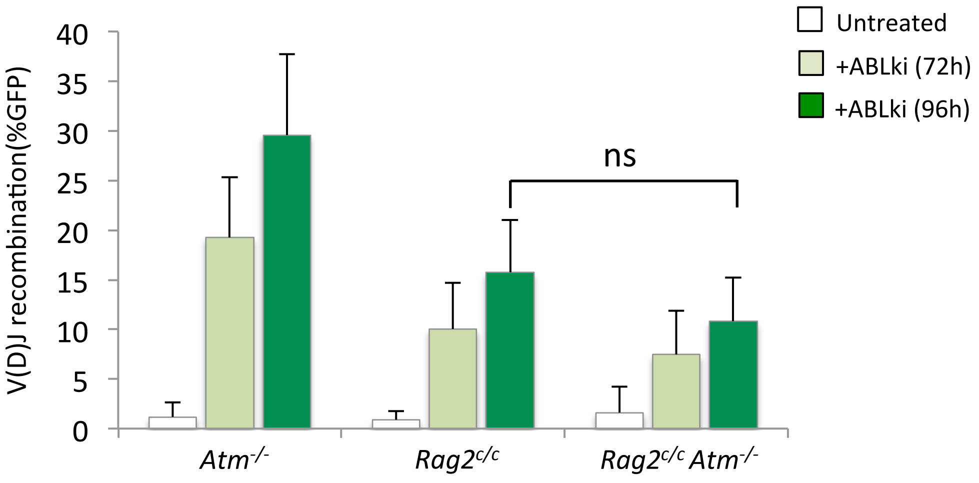

Supplementary Figure 7. Robust pMX-INV rearrangement in *Rag2$^{c/c}$ Atm$^{-/-}$ v-abl* pro-B cells.
v-abl pro–B cell lines of indicated genotypes treated with ABLki for 0, 72h and 96h were assayed for PMX-INV rearrangement by flow cytometry, with the percentage of GFP expressing cells indicated. Histograms represent the means ± SD of 4 *Atm$^{-/-}$* (160, 162, 13560, 13563), 6 *Rag2$^{c/c}$* (56, 145, 12018, 12019, 13570, 13572) and 3 *Rag2$^{c/c}$ Atm$^{-/-}$* (144, 13571, 13573) independent cell lines.

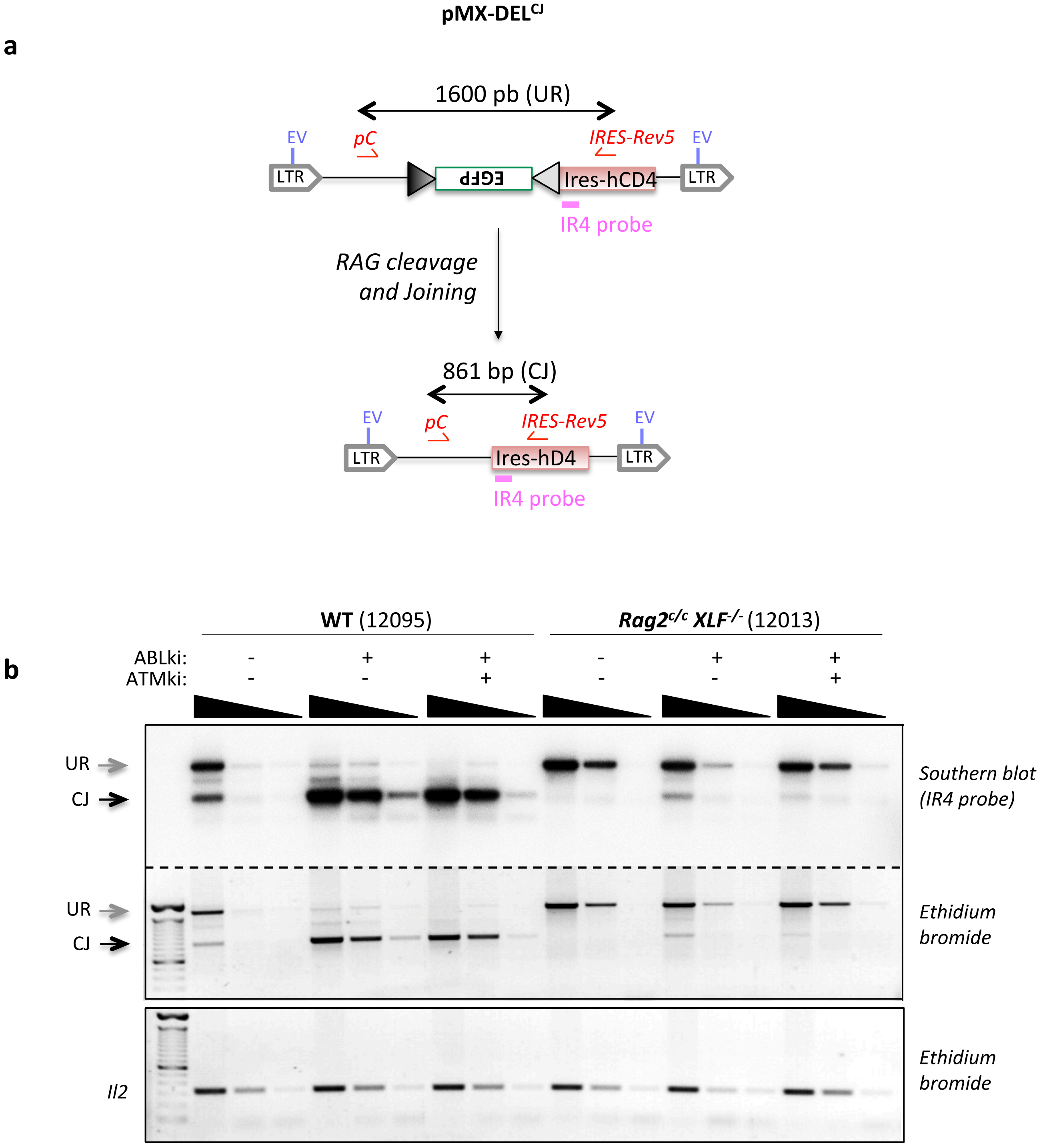

Supplementary Figure 8. PCR analysis of pMX-DEL^{CJ} coding joints in *v-abl* pro-B cell lines.
a. Schematic of PCR approach for analyzing pMX-DEL^{CJ} coding joints (CJs). The pC and IRES REV5 primers used to amplify CJs and the IR4 oligonucleotide probe for Southern blot analysis are indicated. **b.** Southern blot with IR4 probe of pMX-DEL^{CJ} PCR. WT and *Rag2$^{c/c}$ XLF$^{-/-}$ v-abl* pro-B cell lines were treated for 72h with ABLki with or without ATMki. Serial five-fold dilutions of genomic DNA were amplified and run on a 1.2% agarose gel. Ethidium bromide gel (middle panel) has been transferred on a membrane and blotted with IR4 probe (top panel). Il-2 gene PCR was used as a loading control (bottom panel).

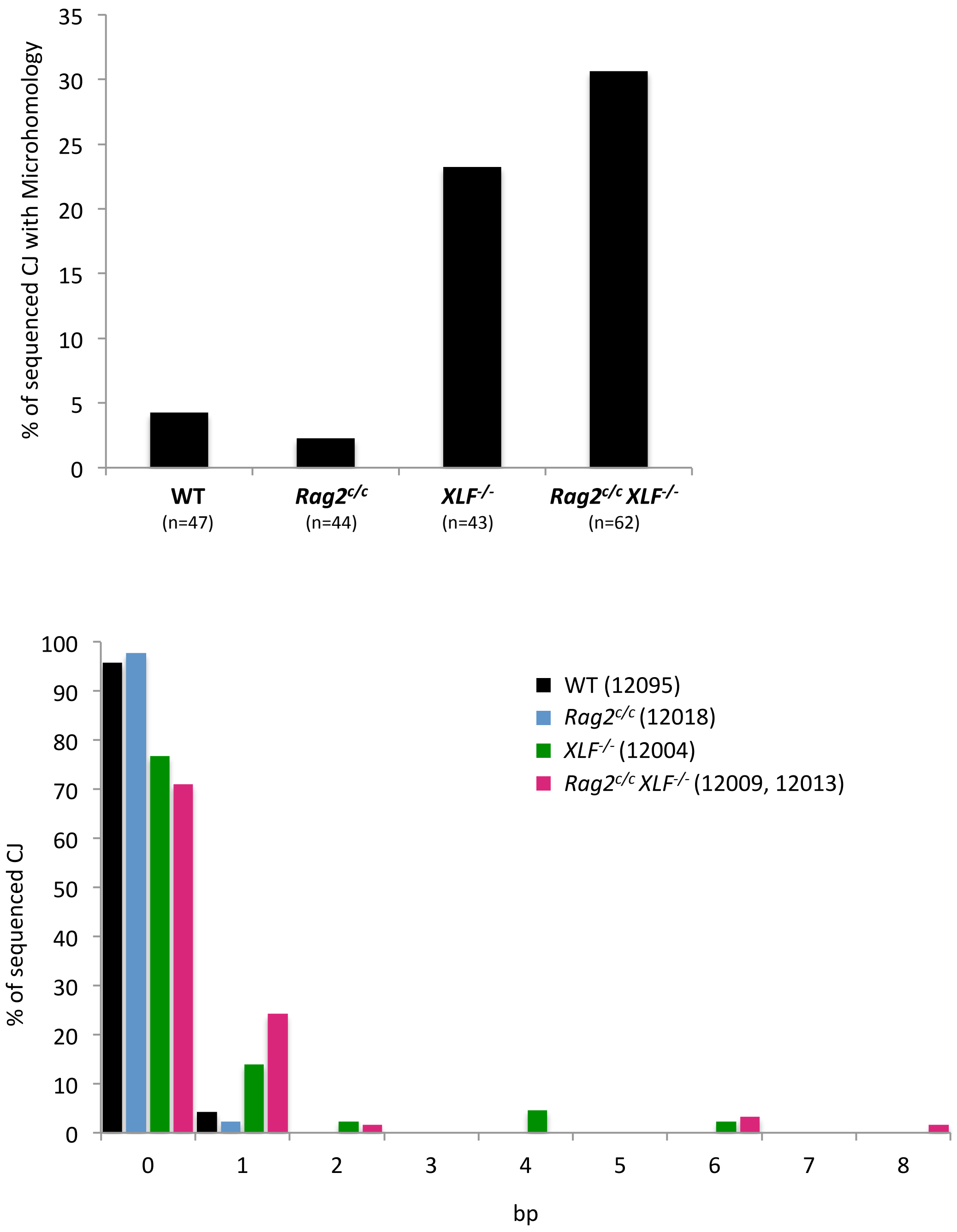

Supplementary Figure 9. Analysis of coding joint sequences in *v-abl* pro-B cell lines.
Microhomology utilization (% of sequences with microhomologies ≥ 1bp, top panel; microhomology length distribution among the same sequences, bottom panel) in pMX-DEL^{CJ} CJs sequenced from indicated *v-abl* pro-B cell lines.

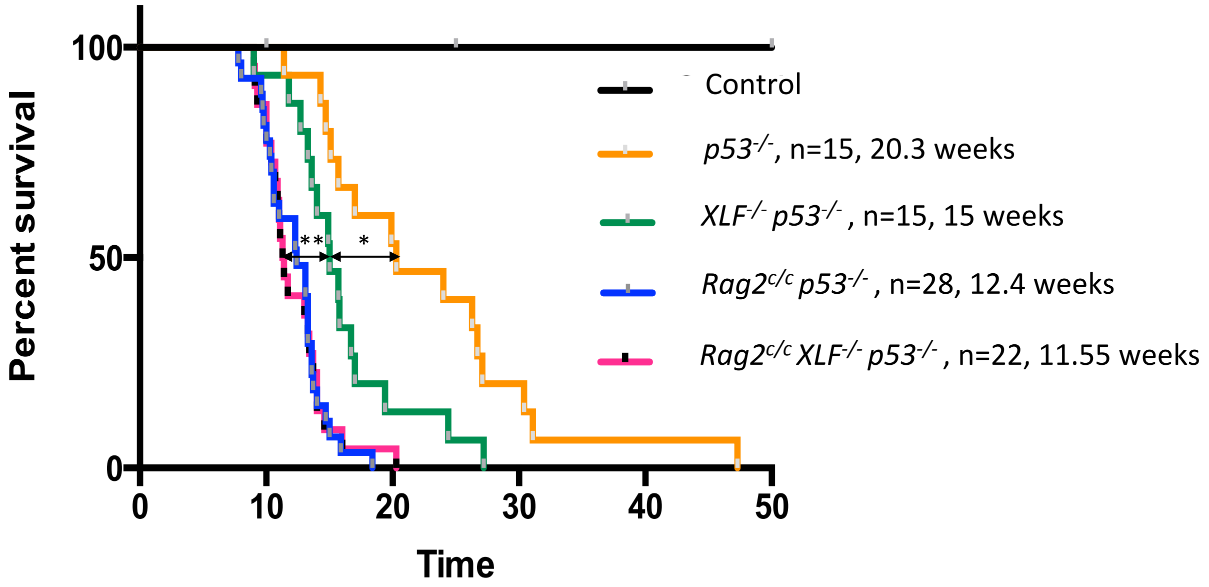

Supplementary Figure 10. Analysis of *Rag2$^{c/c}$ XLF$^{-/-}$ p53$^{-/-}$* mice survival.
Kaplan–Meier survival curves of WT controls, *p53$^{-/-}$* (n=15), *XLF$^{-/-}$ p53$^{-/-}$* (n=15), *Rag2$^{c/c}$ p53$^{-/-}$* (n=28) and *Rag2$^{c/c}$ XLF$^{-/-}$ p53$^{-/-}$* mice (n=22). * $0.01 \le p < 0.05$, ** $0.001 \le p < 0.01$.

Supplementary Figure 11

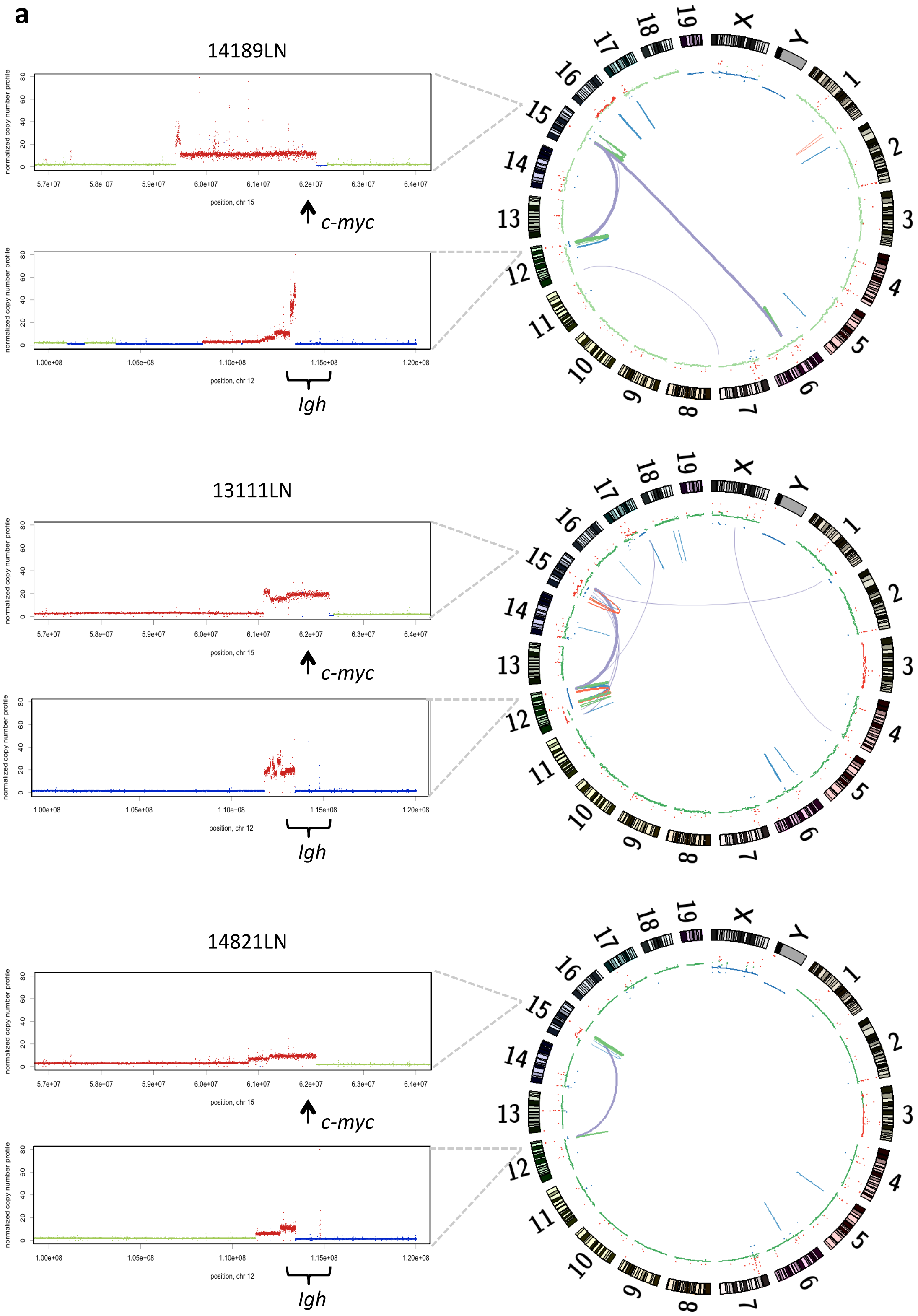

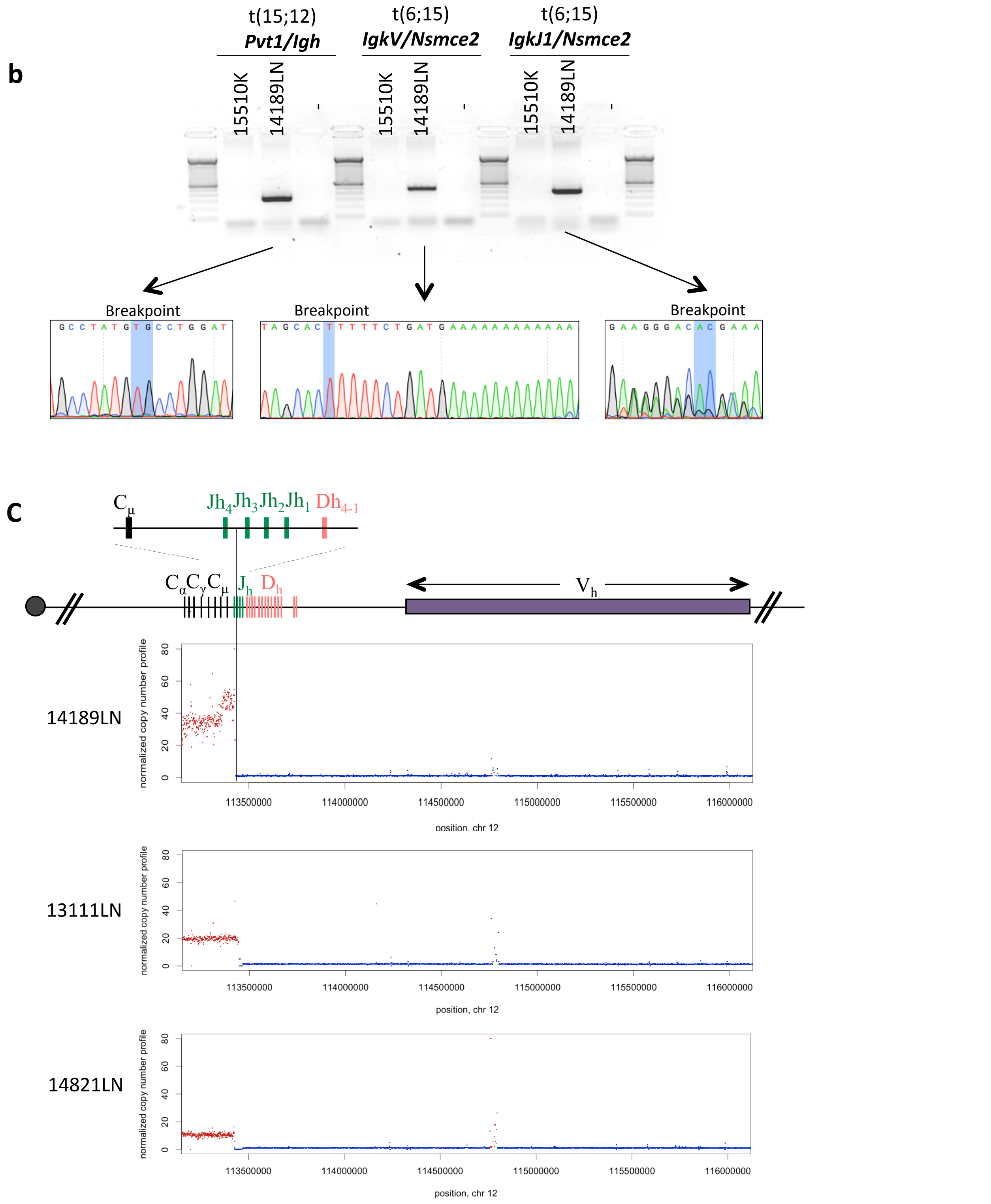

Supplementary Figure 11. Structural variant analysis from WGS of *$Rag2^{c/c}$ $XLF^{-/-}$ $p53^{-/-}$* B lymphomas

a. Circos plots (right panels) showing chromosomal rearrangements from whole genome sequencing of 3 *$Rag2^{c/c}$ $XLF^{-/-}$ $p53^{-/-}$* B lymphomas (# 14189LN, 13111LN and 14821LN). Chromosomes are arranged circularly end-to-end with each chromosome's cytobands marked in the outer ring. The inner ring displays copy number data inferred from WGS with blue dots indicating losses, red dots indicating gains and green dots indicating normal copy numbers. Within circles, purple, orange, green and blue lines represent inter-chromosomal rearrangements, duplications, inversions and deletions, respectively. The thickness of the link is proportional to the number of read pairs supporting the structural variant. Zoom in (left panels) of chromosome 12 (bottom) and 15 (top) indicate copy number changes initiated within the *Igh* locus and including *c-myc*. **b.** PCR amplification and sanger sequencing of the top 3 inter-chromosomal rearrangements identified in the pro-B cell lymphoma #14189LN confirmed the bio-informatically predicted breakpoint locations and presence of microhomologies (See also Supplementary Data 1). **c.** Copy number analysis of the *Igh* locus in B cell tumors #14189LN, 13111LN and 14821LN.

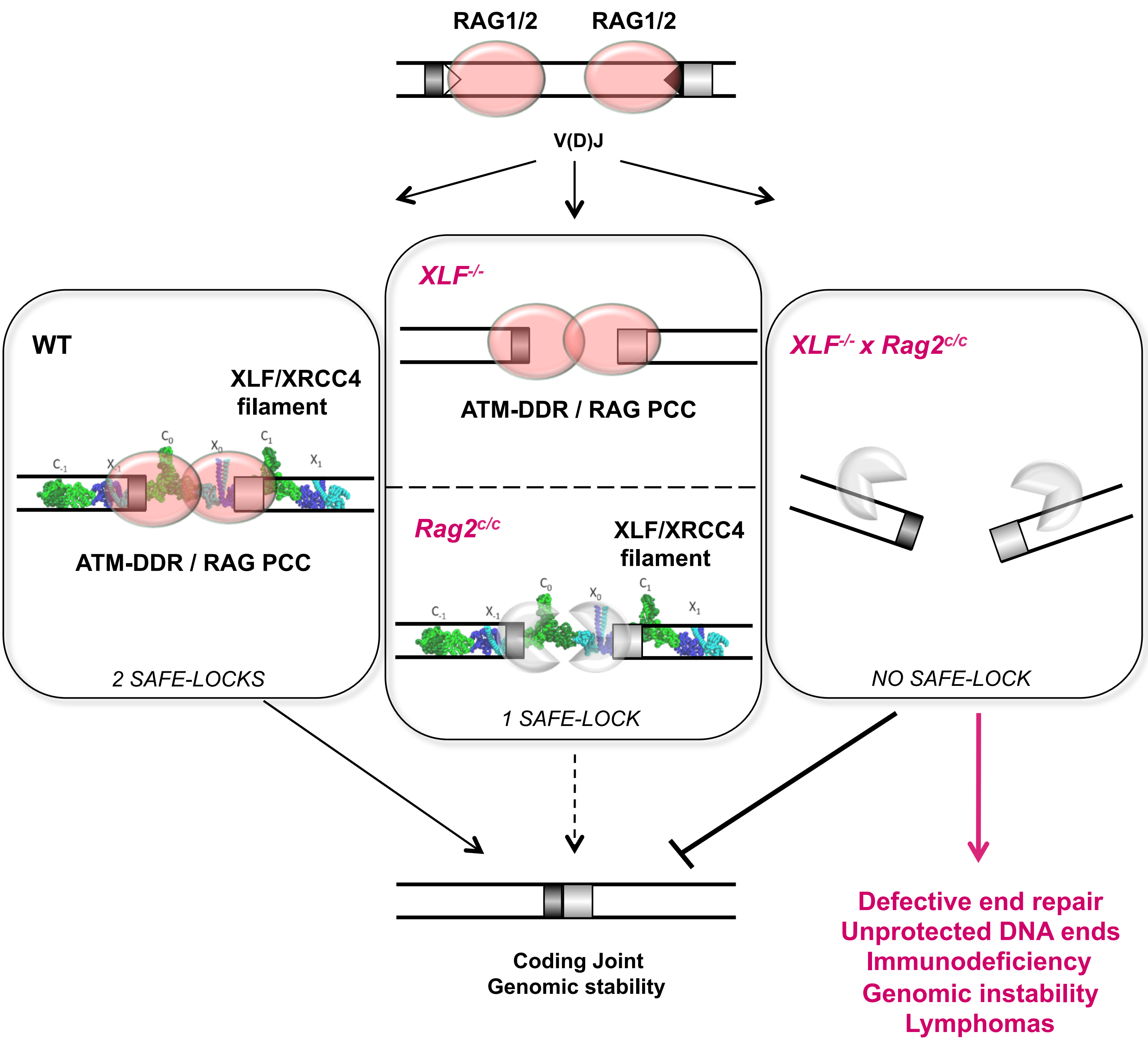

Supplementary Figure 12. A two-tier synapse model for DNA repair during V(D)J recombination.
In wild type lymphocytes (WT), the RAG proteins remain associated with coding ends in a post cleavage complex (RAG PCC), constituting one tier of the synapse (i.e. first safe-lock). The XLF-XRCC4 hetero-filaments appear to help tether broken coding ends and constitute a second tier of the synapse (i.e. second safe-lock). Core RAG2 does not exacerbate the phenotypes associated with ATM deficiency, suggesting that the RAG complex and ATM act in the same pathway (i.e. the ATM-DDR/RAG PCC). When one safe-lock is missing (such as in *Rag2*$^{c/c}$, *XLF*$^{-/-}$, *Atm*$^{-/-}$, *Rag2*$^{c/c}$ *Atm*$^{-/-}$ or *Rag2*$^{c/c}$ cells treated with ATMki), overall DNA end repair is preserved but aberrant hybrid joint formation is observed, indicative of DNA end destabilization. When both safe-locks are missing (such as in *Rag2*$^{c/c}$ *XLF*$^{-/-}$, *XLF*$^{-/-}$*ATM-DDR*$^{-/-}$, or *XLF*$^{-/-}$ cells treated with ATMki), collapse of the synapse leads to the release of DNA ends (which are potentially subjected to ATM-dependent degradation), abortion of V(D)J recombination and genomic instability. We suggest that, in the context of normal physiological antigen receptor assembly, the RAG1/2 endonuclease may have co-evolved with XLF to assist repair of generated DNA breaks through a very efficient double safe-lock mechanism, minimizing the inherent risks of genome instability and neoplastic transformation.

Supplementary Table 1a

		WT				$Rag2^{c/c}$				$XLF^{-/-}$				$Rag2^{c/c}\ XLF^{-/-}$				$Ku80^{-/-}$				$Rag^{-/-}$			
		12095		12096		12018		12019		12004		12005		12009		12013		12070		12071		129M		129F	
	Nb 53BP1 foci/ nucleus	Nuclei Nb	Nuclei %	Nuclei Nb	Nuclei %	Nuclei Nb	Nuclei %	Nuclei Nb	Nuclei %	Nuclei Nb	Nuclei %	Nuclei Nb	Nuclei %	Nuclei Nb	Nuclei %	Nuclei Nb	Nuclei %	Nuclei Nb	Nuclei %	Nuclei Nb	Nuclei %	Nuclei Nb	Nuclei %	Nuclei Nb	Nuclei %
EXPMT 1	0	1728	70.2	2784	67.9	3027	60.5	2992	59.8	2408	48.2	2414	48.1	1827	36.3	1293	47.4	1320	38	ND	ND	ND	ND	ND	ND
	1	589	23.9	1018	24.8	1422	28.4	1485	29.7	2107	42.1	2125	42.3	1887	37.5	899	33	1484	42.7	ND	ND	ND	ND	ND	ND
	2	86	3.5	179	4.4	268	5.4	324	6.5	424	8.5	400	8	1035	20.5	394	14.4	561	16.2	ND	ND	ND	ND	ND	ND
	>2	58	2.3	121	3	287	5.8	206	4	61	1.1	79	1.6	289	5.8	141	5.2	108	3.1	ND	ND	ND	ND	ND	ND
	TOTAL	2461	100	4102	100	5004	100	5007	100	5000	100	5018	100	5038	100	2727	100	3473	100	ND	ND	ND	ND	ND	ND
EXPMT 2	0	4023	66.5	3883	64.5	3433	56.3	3129	52.1	3740	61.8	2977	49.1	1095	26.3	1264	31.7	1466	29.8	ND	ND	ND	ND	ND	ND
	1	1554	25.7	1424	23.7	1744	28.6	1886	31.4	1847	30.5	2293	37.9	1767	42.5	1510	37.9	2435	49.5	ND	ND	ND	ND	ND	ND
	2	214	3.5	278	4.6	375	6.1	444	7.4	337	5.6	492	8.1	977	23.5	744	18.7	843	17.1	ND	ND	ND	ND	ND	ND
	>2	255	4.3	431	7.1	546	8.9	546	9.1	124	1.9	295	4.8	322	7.6	469	11.7	176	3.6	ND	ND	ND	ND	ND	ND
	TOTAL	6046	100	6016	100	6098	100	6005	100	6048	100	6057	100	4161	100	3987	100	4920	100	ND	ND	ND	ND	ND	ND
EXPMT 3	0	4092	68.9	ND	ND	ND	ND	ND	ND	ND	ND	ND	ND	1290	25.9	2165	36.5	ND	ND	ND	ND	5169	85.8	ND	ND
	1	1521	25.6	ND	ND	ND	ND	ND	ND	ND	ND	ND	ND	2233	44.9	2227	37.5	ND	ND	ND	ND	476	7.9	ND	ND
	2	203	3.4	ND	ND	ND	ND	ND	ND	ND	ND	ND	ND	1172	23.6	1211	20.4	ND	ND	ND	ND	129	2.1	ND	ND
	>2	126	2.2	ND	ND	ND	ND	ND	ND	ND	ND	ND	ND	280	5.6	328	5.6	ND	ND	ND	ND	247	4	ND	ND
	TOTAL	5942	100	ND	ND	ND	ND	ND	ND	ND	ND	ND	ND	4975	100	5931	100	ND	ND	ND	ND	6021	100	ND	ND
EXPMT 4	0	4067	67.6	3637	60.3	4032	67.2	3298	54.6	1817	30	ND	ND	1820	30.3	2367	39.1	ND	ND	ND	ND	5351	88.8	5304	86.8
	1	1775	29.5	2148	35.6	1697	28.3	2319	38.4	3154	52.1	ND	ND	2535	42.2	2143	35.4	ND	ND	ND	ND	561	9.3	365	6
	2	147	2.4	220	3.6	232	3.9	387	6.4	917	15.1	ND	ND	1332	22.2	1182	19.6	ND	ND	ND	ND	66	1.1	118	1.9
	>2	31	0.4	26	0.4	42	0.7	41	0.6	168	2.7	ND	ND	323	5.3	354	5.8	ND	ND	ND	ND	50	0.8	322	5.1
	TOTAL	6020	100	6031	100	6003	100	6045	100	6056	100	ND	ND	6010	100	6046	100	ND	ND	ND	ND	6028	100	6109	100
EXPMT 5	0	ND	ND	ND	ND	4083	67.8	3019	49.7	2318	38.6	ND	ND	ND	ND	ND	ND	ND	ND	1620	26.9	ND	ND	ND	ND
	1	ND	ND	ND	ND	1467	24.3	2332	38.4	2893	48.2	ND	ND	ND	ND	ND	ND	ND	ND	2574	42.7	ND	ND	ND	ND
	2	ND	ND	ND	ND	252	4.2	496	8.2	676	11.3	ND	ND	ND	ND	ND	ND	ND	ND	1441	23.9	ND	ND	ND	ND
	>2	ND	ND	ND	ND	224	3.6	229	3.8	118	1.9	ND	ND	ND	ND	ND	ND	ND	ND	395	6.5	ND	ND	ND	ND
	TOTAL	ND	ND	ND	ND	6026	100	6076	100	6005	100	ND	ND	ND	ND	ND	ND	ND	ND	6030	100	ND	ND	ND	ND

Supplementary Table 1b

	WT		$Rag2^{c/c}$		$XLF^{-/-}$		$Rag2^{c/c}\ XLF^{-/-}$		$Ku80^{-/-}$		$Rag^{-/-}$	
Nb 53BP1 foci/ nucleus	Mean	SD	Mean	SD	Mean	SD	Mean	SD	Mean	SD	Mean	SD
0	66.6	3.3	58.5	6.6	46.0	10.8	34.2	7.2	31.6	5.8	87.1	1.5
1	27.0	4.3	30.9	5.0	42.2	7.6	38.9	4.0	45.0	3.9	7.7	1.7
2	3.6	0.7	6.0	1.5	9.4	3.3	20.4	3.0	19.1	4.2	1.7	0.5
>2	2.8	2.3	4.6	3.2	2.3	1.3	6.6	2.2	4.4	1.8	3.3	2.2
Total nuclei		36618		46264		34184		38875		14423		18158

Supplementary Table 1. *$Rag2^{c/c}$ $XLF^{-/-}$ v-abl* pro–B cell harbor increased 53BP1 foci after RAG induction.
a. Table indicates the number and percentage of *v-abl* pro-B cells harboring 0, 1, 2 or >2 53BP1 foci 65h after ABLki treatment. 5 independent experiments are shown. **b**. Table indicates the mean and SD of the experiments detailed in table **a**.

Supplementary Table 2a

Seq Nb	5'CE	MH	P/N	3'CE
	TAATAGGAACGTCTG			CAGCCTACAATTCCG
1	TAATAGGAACGTCTG			CAGCCTACAATTCCG
2	TAATAGGAACGTCT		TAGG	GCCTACAATTCCG
3	TAATAGGAACG		G	CAGCCTACAATTCCG
4	TAATAGGAACGTCTG			CAGCCTACAATTCCG
5	TAATAGGAACGTCTG			CAATTCCG
6	TAATAGGAACGTCTG			CAGCCTACAATTCCG
7	TAATAGGAACGTCT		A	AGCCTACAATTCCG
8	TAATAGGAACGTCTG			CAATTCCG
9	TAATAGGAACGTCT			CAATTCCG
10	TAATAGGAACGTCT		A	AGCCTACAATTCCG
11	TAATAGGAACGT		TA	ACAATTCCG
12	TAATAGGAACG		ACCG	CAGCCTACAATTCCG
13	TAATAGGAACGTCTG			CAGCCTACAATTCCG
14	TAATAGGAACGTCTG		AG	AGCCTACAATTCCG
15	TAATAGGAACGTCT			CCTACAATTCCG
16	TAATAGGAACGTC		CCCTG	CAGCCTACAATTCCG
17	TAATAGGAACGTCTG		C	CAGCCTACAATTCCG
18	TAATAGGAACGTCTG			CCTACAATTCCG
19	TAATAGGAACGTCTG			CAGCCTACAATTCCG
20	TAATAGGAACGTCTG			AGCCTACAATTCCG
21	TAATAGGAACG		CCC	CAGCCTACAATTCCG
22	TAATAGGAACGTCTG			CAGCCTACAATTCCG
23	TAATAGGAACGT		G	CAGCCTACAATTCCG
24	TAATAGGAACGTC		CCT	CAGCCTACAATTCCG
25	TAATAGGAACG		G	CAGCCTACAATTCCG
26	TAATAGGAACGTCT	G		CCTACAATTCCG
27	TAATAGGAACGTCTG			CAGCCTACAATTCCG
28	TAATAGGAACGTCTG			CAGCCTACAATTCCG
29	TAATAGGAACGTCT		AGGG	ATTCCG
30	TAATAGGAACG			CAGCCTACAATTCCG
31	TAATAGGAACGTCTG			CAGCCTACAATTCCG
32	TAATAGGAACGTCTG			ACCTACAATTCCG
33	TAATAGGAACGTC		CC	CAGCCTACAATTCCG
34	TAATAGGAACGT		G	CAGCCTACAATTCCG
35	TAATAGGAACGTCT		AA	AGCCTACAATTCCG
36	TAATAGGAACGT			ACAATTCCG
37	TAATAGGAACGTCTG			AGCCTACAATTCCG
38	TAATAGGAAC		CCCCCTG	CAGCCTACAATTCCG
39	TAATAGGAACGTCTG			CAGCCTACAATTCCG
40	TAATAGGAACG		G	CAGCCTACAATTCCG
41	TAATAGGAACGTCT	G		CCTACAATTCCG
42	TAATAGGAACG		G	CAGCCTACAATTCCG
43	TAATAGGAACGTCT		A	AGCCTACAATTCCG
44	TAATAGGAACGTCTG			CAGCCTACAATTCCG
45	TAATAGGAACGT		TCT	ATTCCG
46	TAATAGGAACGTCTG			CAGCCTACAATTCCG
47	TAATAGGAACGTC		G	CAGCCTACAATTCCG

Supplementary Table 2. Coding joint sequences from WT:DEL^{CJ} (a) and *Rag2$^{c/c}$ XLF$^{-/-}$* :DEL^{CJ} (b) *v-abl* pro-B cells. The reference sequence for 5' and 3' coding ends generated from the pMX-DEL^{CJ} substrate is shown at the top. When coding ends are resected beyond the 15 nucleotides shown in the reference sequence, the number of nucleotides resected is indicated in brackets along with the reference sequence flanking the resected end (in italics). N- and P- nucleotide additions and microhomologies (MH) are indicated. * For sequences n° 7 and 35, the coding joints are located 205 and 166 bp downstream of RSS-12 respectively, potentially corresponding to a miscleavage or a single cut at the RSS-23 followed by extensive resection. UR: unrearranged sequence.

Supplementary Table 2b

Seq Nb		5'CE	MH	P/N	3'CE	
		TAATAGGAACGTCTG			CAGCCTACAATTCCG	
1		TAATAGGAA	C		CTACAATTCCG	
2		TAATAGGAACGT		G	CAGCCTACAATTCCG	
3		TAATAGGAACGT	C		G	
4		TAATAGGAACG			CCG	
5		TAATAGGAACG			ACAATTCCG	
6		TAATAGGAACGTCTG			CAGCCTACAATTCCG	
				GCACGGGGCCGTCGCC		*CTGGGGCCTCGGTGCA*
7*		TAATAGGAACGTCTG		**205bp UR**-GCACGGGGCC	(-478)	TCGGTGCA
8		TAATAGGAACGTCT			AGCCTACAATTCCG	
9		TAATAGGAACGTC			ATTCCG	
10		TAATAG		AAAC	GCCTACAATTCCG	
		TAATAGGAACGTCTG				*ACAACGTCTGTAGCGACC*
11		TAATAGG	AACGTCTG		(-232)	TAGCGACC
	TCTGGTGTCAAA					*CGCCCCCCCCCCT*
12	TCTGGTGT	(-6)		T	(-2)	CCCCCCCCT
13		TAATAGGAACG			CCG	
	CTGGTGTCAAAAA				*CAGCCTACAATTCCG*	
14	CTGGTGTC	(-5)			GCCTACAATTCCG	
15		TAATAGGAACGTC		C	CAGCCTACAATTCCG	
16		TAATAGGAACGTCT			CCG	
17		TAATAGGAACGTCTG			CAGCCTACAATTCCG	
18		TAATAGGAACGTCT	G		CCTACAATTCCG	
	GTGTCAAAAA					
19	GTGTCAAA	(-2)		G	CG	
20		TAATAGGAACG	T		ACAATTCCG	
21		TAATAGGAACG	T		TCCG	
22		TAATAGGAACGTC	T		TCCG	
23		TAATAGGAACG	T		ACAATTCCG	
24		TAATAGGAACG		G	CAGCCTACAATTCCG	
25		TAATAGGAACG		GG	CAGCCTACAATTCCG	
	GTCCGCCGACAC					*AAGCCACGTGTA*
26	GTCCGCCG	(-232)			(-303)	CACGTGTA
27		TAATAGGAACGTCTG		CAAA	CTACAATTCCG	
28		TAATAGGAACGT		T	ATTCCG	
29		TAATAGGAACGTC	T		TCCG	
30		TAATAGGAACGTCTG			CAGCCTACAATTCCG	
31		TAATAGG		GACGTG	CAGCCTACAATTCCG	
32		TAATAGGAACGTC		G	CAGCCTACAATTCCG	
33		TAATAGGAACGTCTG		A	AGCCTACAATTCCG	
34		TAATAGGAACGTCTG			CAGCCTACAATTCCG	
				GGGCGGACTGGGTGCT		*ACAACCCCAGTGCCA*
35*		TAATAGGAACGTCTG		**166bp UR**-GGGCGGACTG	(-356)	CAGTGCCA
36		TAATAGGAACG			AATTCCG	
	ATTACCAACTTG					*ATAAGGCCGGTG*
37	ATTACC	(-18)	AA		(-42)	GGCCGGTG
38		TAATAGGAACG	T		TCCG	
	ACCACCCCCACC					*GCTTCTTGAAGA*
39	ACCACCCC	(-168)		**39bp insertion**	(-218)	CTTGAAGA
	TACCAACTTGTC					*CCGCCCCCCCCCCT*
40	TACCAACT	(-16)			(-1)	CCCCCCCCT
41		TAATAGGAACGTC		G	CAGCCTACAATTCCG	
42		TAATAGGAACGTC			CAATTCCG	
43		TAATAGGAACGT	C		AGCCTACAATTCCG	
44		TAATAGGAACG		A	AATTCCG	
45		TAATAGGAACG	T		TCCG	
	GTCCGCCGACAC					*AAGCCACGTGTA*
46	GTCCGCCG	(-232)			(-303)	CACGTGTA
47		TAATAGGAACG		A	CCTACAATTCCG	
48		TAATAGGAACG		CAG	CAGCCTACAATTCCG	
	GTCCGCCGACAC					*AAGCCACGTGTA*
49	AGTCCTGC	(-179)			(-406)	CAAGGGGC
50		TAATAGGAACG		A	CCTACAATTCCG	
51		TAATAGGAACG			CAGCCTACAATTCCG	
52		TAATAGGAACG	T		TCCG	
53		TAATAGGAACGTCTG			CAGCCTACAATTCCG	
54		TAATAGGAACG	T		TCCG	
						CCTAACGTTACT
55		TAATAGGAACGTCT			(-12)	ACGTTACT
56		TAA	T		TCCG	
57		TAATAGGAACGTCTG			AGCCTACAATTCCG	
	TGCTGACCACCCCCAC					*GAACCCCCCACCTGGC*
58	TGCTGACC	(-168)	ACCCCC		(-264)	CACCTGGC
59		TAATAGGAACGTCTG			AGCCTACAATTCCG	
60		TAATAGGAACG	T		TCCG	
61		TAATAGGAACGTCTG			AGCCTACAATTCCG	
	GTCCGCCGACAC					*AAGCCACGTGTA*
62	GTCCGCCG	(-232)			(-303)	CACGTGTA

Supplementary Table 3a

Genotype	Cell line #	Untreated					
		Metaphases with chromosome breaks	Metaphases with translocations	Total aberrant metaphases	Total normal metaphases	Total metaphases analyzed	% aberrant metaphases
**WT**	12095	3	0	3	202	205	1.5
	12096 (1)	1	0	1	198	199	0.5
	12096 (2)	0	0	0	81	81	0
	165	1	0	1	202	203	0.5
	**TOTAL**	**5**	**0**	**5**	**683**	**688**	**0.7**
***Rag2**$^{c/c}$	12018 (1)	2	0	2	198	200	1.0
	12019	0	0	0	79	79	0.0
	12018 (2)	5	0	5	384	389	1.3
	13572	5	0	5	95	100	5.0
	**TOTAL**	**12**	**0**	**12**	**756**	**768**	**1.6**
***XLF**$^{-/-}$	12004	2	4	6	214	220	2.7
	12005	2	0	2	164	166	1.2
	**TOTAL**	**4**	**4**	**8**	**378**	**386**	**2.1**
***Rag2**$^{c/c}$ ***XLF**$^{-/-}$	12013	2	0	2	208	210	1.0
	12009	0	0	0	200	200	0.0
	14837	ND	ND	ND	ND	ND	ND
	**TOTAL**	**2**	**0**	**2**	**408**	**410**	**0.5**
***p53**$^{-/-}$	6920.1	ND	ND	ND	ND	ND	ND
	6943.2	ND	ND	ND	ND	ND	ND
	15307	4	4	8	122	130	6.2
	**TOTAL**	**4**	**4**	**8**	**122**	**130**	**6.2**
***Rag2**$^{c/c}$ ***p53**$^{-/-}$	4566.7	ND	ND	ND	ND	ND	ND
***XLF**$^{-/-}$ ***p53**$^{-/-}$	15259	9	1	10	190	200	5
	15293	4	3	7	156	163	4.3
	**TOTAL**	**13**	**4**	**17**	**346**	**363**	**4.7**
***Rag2**$^{c/c}$ ***XLF**$^{-/-}$ ***p53**$^{-/-}$	14838	5	2	7	253	260	2.7
	14840	18	5	23	277	300	7.7
	**TOTAL**	**23**	**7**	**30**	**530**	**560**	**5.4**
***Rag**$^{-/-}$ ***p53**$^{-/-}$	6527.2	ND	ND	ND	ND	ND	ND

Supplementary Table 3b

Genotype	Cell line #	PD180970-treated and released: Metaphases with chromosome breaks	Metaphases with translocations	Total aberrant metaphases	Total normal metaphases	Total metaphases analyzed	% aberrant metaphases
**WT**	12095	2	4	6	199	205	2.9
	12096 (1)	5	2	7	198	205	3.4
	12096 (2)	8	2	10	378	388	2.6
	165	7	7	14	369	383	3.7
	**TOTAL**	**22**	**15**	**37**	**1144**	**1181**	**3.1**
Rag2$^{c/c}$	12018 (1)	3	4	7	203	210	3.3
	12019	4	9	13	187	200	6.5
	12018 (2)	12	7	19	364	383	5.0
	13572	8	6	14	86	100	14.0
	**TOTAL**	**27**	**26**	**53**	**840**	**893**	**5.9**
XLF$^{-/-}$	12004	7	8	15	185	200	7.5
	12005	4	2	6	186	192	3.1
	**TOTAL**	**11**	**10**	**21**	**371**	**392**	**5.4**
Rag2$^{c/c}$ XLF$^{-/-}$	12013	0	5	5	405	410	1.2
	12009	2	4	6	506	512	1.2
	14837	0	1	1	119	120	0.8
	**TOTAL**	**2**	**10**	**12**	**1030**	**1042**	**1.2**
p53$^{-/-}$	6920.1	7	2	9	241	250	3.6
	6943.2	8	8	16	235	251	6.4
	15307	7	13	20	224	244	8.2
	**TOTAL**	**22**	**23**	**45**	**700**	**745**	**6.0**
Rag2$^{c/c}$ p53$^{-/-}$	4566.7	**12**	**8**	**20**	**230**	**250**	**8.0**
XLF$^{-/-}$ p53$^{-/-}$	15259	1	9	10	196	206	4.9
	15293	3	10	13	184	197	6.6
	**TOTAL**	**4**	**19**	**23**	380	**403**	**5.7**
Rag2$^{c/c}$ XLF$^{-/-}$ p53$^{-/-}$	14838	17	65	82	283	365	22.5
	14840	13	33	46	313	359	12.8
	**TOTAL**	**30**	**98**	**128**	**596**	**724**	**17.7**
Rag$^{-/-}$ p53$^{-/-}$	6527.2	0	0	0	250	250	0.0

Statistical analysis (Fisher exact test)		p value	Level
Rag2$^{c/c}$ XLF$^{-/-}$ p53$^{-/-}$	vs *XLF$^{-/-}$ p53$^{-/-}$*	3.13e-9	***
	vs *Rag2$^{c/c}$ p53$^{-/-}$*	1.43e-4	***
	vs *p53$^{-/-}$*	2.22e-12	***
Rag2$^{c/c}$ XLF$^{-/-}$	vs *XLF$^{-/-}$*	1.11e-5	***
	vs *Rag2$^{c/c}$*	6.71e-5	***
	vs WT	0.012	*
XLF$^{-/-}$	vs WT	0.16	ns
Rag2$^{c/c}$	vs WT	0.29	ns

Supplementary Table 3. Genomic instability at the *Igk* locus in *v-abl* pro-B cells.
Number and percentage of aberrant metaphases harboring chromosomes breaks and/or translocations involving the *Igk* locus from untreated (**a**) and ABLki-treated and released (**b**) *v-abl* pro-B cell lines of the indicated genotype are indicated. DNA FISH experiments were performed using probes centromeric (*Igk V*) and telomeric (*Igk C*) to the *Igk* locus plus specific paint for chromosome 6.

Genotype	Experiment	Metaphases with chromosome breaks	Metaphases with translocations	Total aberrant metaphases	Total normal metaphases	Total metaphases analyzed	% aberrant metaphases
**WT**	Expriment 1	0	0	0	79	79	0.0
	Expriment 2	0	0	0	122	122	0.0
	**TOTAL**	**0**	**0**	**0**	**201**	**201**	**0.0**
Rag2$^{c/c}$	Expriment 1	0	0	0	167	167	0.0
	Expriment 2	1	0	1	116	117	0.9
	Expriment 3	0	0	0	310	310	0.0
	**TOTAL**	**1**	**0**	**1**	**593**	**594**	**0.2**
XLF$^{-/-}$	Expriment 1	0	0	0	108	108	0.0
	Expriment 2	1	0	1	304	305	0.3
	Expriment 3	1	0	1	134	135	0.7
	**TOTAL**	**2**	**0**	**2**	**546**	**548**	**0.4**
Rag2$^{c/c}$ XLF$^{-/-}$	Expriment 1	0	0	0	141	141	0.0
	Expriment 2	1	0	1	99	100	1.0
	Expriment 3	0	1	1	219	220	0.5
	Expriment 4	0	0	0	300	300	0.0
	**TOTAL**	**1**	**1**	**2**	**759**	**761**	**0.3**
p53$^{-/-}$	Expriment 1	3	0	3	114	117	2.6
	Expriment 2	2	0	2	102	104	1.9
	Expriment 3	2	0	2	198	200	1.0
	**TOTAL**	**7**	**0**	**7**	**414**	**421**	**1.7**
Rag2$^{c/c}$ p53$^{-/-}$	Expriment 1	3	0	3	143	146	2.1
	Expriment 2	5	0	5	145	150	3.3
	Expriment 3	8	1	9	268	277	3.2
	Expriment 4	13	1	14	296	310	4.5
	**TOTAL**	**29**	**2**	**31**	**852**	**883**	**3.5**
XLF$^{-/-}$ p53$^{-/-}$	Expriment 1	3	0	3	297	300	1.0
	Expriment 2	13	0	13	187	200	6.5
	Expriment 3	15	0	15	195	210	7.1
	Expriment 4	7	0	7	193	200	3.5
	**TOTAL**	**38**	**0**	**38**	**872**	**910**	**4.2**
Rag2$^{c/c}$ XLF$^{-/-}$ p53$^{-/-}$	Expriment 1	17	9	26	218	244	10.7
	Expriment 2	23	7	30	259	289	10.4
	Expriment 3	15	2	17	193	210	8.1
	Expriment 4	19	5	24	176	200	12.0
	**TOTAL**	**74**	**23**	**97**	**846**	**943**	**10.3**

**Statistical analysis (Fisher exact test)**		p value	Level
Rag2$^{c/c}$ XLF$^{-/-}$ p53$^{-/-}$	vs *XLF$^{-/-}$ p53$^{-/-}$*	3.92e-7	***
	vs *Rag2$^{c/c}$ p53$^{-/-}$*	7.72e-9	***
	vs *p53$^{-/-}$*	8.55e-10	***
XLF$^{-/-}$ p53$^{-/-}$	vs *p53$^{-/-}$*	0.021	*
	vs *Rag2$^{c/c}$ p53$^{-/-}$*	0.539	ns
Rag2$^{c/c}$ p53$^{-/-}$	vs *p53$^{-/-}$*	0.077	ns

Supplementary Table 4. Genomic instability at the *Igh* locus in primary pro-B cells.
Number and percentage of aberrant metaphases harboring chromosomes breaks and/or translocations involving the *Igh* locus from cultured $CD19^{+}IgM^{-}$ pro-B cell of the indicated genotype are indicated. DNA FISH experiments were performed using probes centromeric (*Igh V*) and telomeric (*Igh C*) to the *Igh* locus plus specific paint for chromosome 12.

Tumor #	Genotype	Immunophenotype	Translocations	Frequency
**629T**	*XLF$^{-/-}$ p53$^{-/-}$*	$CD4^-$ $CD8^+$ (>50% $CD3^+TCR\beta^+$)	t(8;4)	10/10
**722T**	*XLF$^{-/-}$ p53$^{-/-}$*	$CD4^-$ $CD8^+$ (>50% $CD3^+TCR\beta^+$)	-	
**916T**	*XLF$^{-/-}$ p53$^{-/-}$*	$CD4^+CD8^+$ (<50% $CD3^+TCR\beta^+$)	-	
**12415T**	*XLF$^{-/-}$ p53$^{-/-}$*	$CD4^+CD8^-$ (<50% $CD3^+TCR\beta^+$)	t(16;15)	14/15
		and $CD4^+CD8^+$ (<50% $CD3^+TCR\beta^+$)	t(17;7)	14/15
**13348T**	*XLF$^{-/-}$ p53$^{-/-}$*	$CD4^+CD8^+$ (<10% $CD3^+TCR\beta^+$)	(t(X;5)	8/9
**618T**	*Rag2$^{c/c}$ XLF$^{-/-}$ p53$^{-/-}$*	$CD4^+CD8^+$ (>50% $CD3^+TCR\beta^+$)	t(9;1;15)	6/16
			t(9;1)	6/16
			t(19;16)	6/16
			t(14;X)	1/16
**816T**	*Rag2$^{c/c}$ XLF$^{-/-}$ p53$^{-/-}$*	$CD4^+CD8^+$ ($CD3^{lo}TCR\beta^{lo}$)	t(12;2)	13/23
			t(15;2;15)	10/23
			t(12;6)	3/23
			t(12;14)	4/23
			t(12;9)	2/23
			t(14;8)	1/23
**854T**	*Rag2$^{c/c}$ XLF$^{-/-}$ p53$^{-/-}$*	$CD4^+CD8^+$ (<10% $CD3^+TCR\beta^+$)	t(5;3)	14/24
			t(13;16)	3/24
			t(12;17)	2/24
**12534T**	*Rag2$^{c/c}$ XLF$^{-/-}$ p53$^{-/-}$*	$CD4^+CD8^+$ (50% $CD3^+TCR\beta^+$)	t(1;14)	10/33
			t(2;11)	5/33
			t(12;14)	3/33
			t(9;5)	3/33
			t(11;12)	1/33
			t(14;11).(11.14)	1/33
			t(11;16)	1/33
			t(2;Y)	1/33
			t(5;10).(10;5)	1/33
			t(14?;4)	1/33
**12697T**	*Rag2$^{c/c}$ XLF$^{-/-}$ p53$^{-/-}$*	$CD4^+CD8^+$ (<10% $CD3^+TCR\beta^+$)	t(19.14)	8/26
			t(14;19;14)	5/26
			t(12;8)	8/26
			t(12;5)	6/26
			t(12;14)	1/26
			t(12;11)	1/26
			t(12;2)	1/26
			t(12;3)	1/26
			t(17;12?)	1/26
			t(7;16).(16;7)	1/26

Supplementary Table 5. Immunophenotyping and spectral karyotyping of T-cell lymphomas.

Tumor #	Genotype	Immunophenotype	Translocations	Frequency
**14705LN**	*$XLF^{-/-}$ $p53^{-/-}$*	$B220^+CD19^+CD43^-IgM^-$	t(12;5)	20/25
			t(3;12;5)	12/25
			t(12;3)	5/25
			t(3;5)	2/25
			t(3;12)	2/25
			t(13;12;9)	1/25
			t(13;12)	1/25
			dicentric t(1;4)	1/25
**13111LN**	*$Rag2^{c/c}$ $XLF^{-/-}$ $p53^{-/-}$*	$B220^+CD19^+CD43^+IgM^-$	t(1;15)	20/22
			t(12;15;12)	9/22
			t(15;12)	3/22
			t(15;12;6)	3/22
			t(6;12;6)	2/22
			t(17;12)	2/22
			t(15;12;4)	2/22
			t(7;2)	1/22
			t(17;3)	1/22
			t(15;12;17)	1/22
			t(15;12;3)	1/22
			t(3;15)	1/22
			t(15;12;7)	1/22
			t(7;12;16)	1/22
**13519LN**	*$Rag2^{c/c}$ $XLF^{-/-}$ $p53^{-/-}$*	$B220^+CD19^+CD43^+IgM^-$	t(12;15)	20/20
			t(15;12;15)	11/20
			t(15;12)	6/20
			t(15;12;6)	2/20
			t(15;12;2)	1/20
			t(15;12;1)	1/20
**14181LN**	*$Rag2^{c/c}$ $XLF^{-/-}$ $p53^{-/-}$*	$B220^+CD19^+CD43^+IgM^-$	t(12;15;12;7)	6/20
			t(6;7)	5/20
			t(12;15;12;16)	3/20
			t(15;12;15)	2/20
			t(12;15)	2/20
			t(12;15;12;15)	1/20
			t(12;15;12;6)	1/20
			t(6;12;15)	1/20
			t(12;15;12;19)	1/20
			t(5;15?;12;9)	1/20
			dicentrique 6	1/20
			dicentrique 10	1/20
			t(2;12;15;12)	1/20
**14189LN**	*$Rag2^{c/c}$ $XLF^{-/-}$ $p53^{-/-}$*	$B220^+CD19^+CD43^+IgM^-$	t(12;15)	29/30
			t(15;12;15;16)	5/30
			t(15;12;15)	4/30
			t(6;16)	3/30
			t(15;12;15;14)	3/30
			t(15;12;16)	3/30
			t(15;6)	2/30
			t(15;12;6)	2/30
			t(15;12)	2/30
			t(15;12;3)	2/30
			t(6;14)	1/30
			dicentric 6	1/30
			t(15;16)	1/30
			t(15;12;15;3)	1/30
			t(15;12;5)	1/30
			t(17;8)	1/30
			t(15;12;15;18)	1/30
			t(15;19)	1/30
**14821LN**	*$Rag2^{c/c}$ $XLF^{-/-}$ $p53^{-/-}$*	$B220^+CD19^+CD43^+IgM^-$	t(12;15)	20/21
			t(15;12;15)	13/21
			t(15;12;15;6)	5/21
			t(15;12;15;12)	2/21

Supplementary Table 6. Immunophenotyping and spectral karyotyping of B-cell lymphomas.
